# Diseases of the musculoskeletal system and connective tissue and risk of breast cancer: Mendelian randomization study in European and East Asian populations

**DOI:** 10.3389/fonc.2023.1170119

**Published:** 2023-04-26

**Authors:** Yue-chen Xu, Jian-xiong Wang, Yi-ran Chu, Han Qian, Hong-yan Wang, Fan Wang

**Affiliations:** ^1^ Department of Radiotherapy, The First Affiliated Hospital of Anhui Medical University, Hefei, China; ^2^ Department of Rheumatology and Immunology, The First Affiliated Hospital of Anhui Medical University, Hefei, China

**Keywords:** connective tissue disease, breast cancer, European, East Asian, Mendelian randomization

## Abstract

**Objective:**

Associations between diseases of the musculoskeletal system and connective tissue (MSCTD) and breast cancer (BC) have not been elucidated completely. The purpose of this study was to investigate the associations of MSCTD, rheumatoid arthritis (RA), Sjogren syndrome (SS), systemic lupus erythematosus (SLE), systemic sclerosis (SSc), dermatomyositis (DM), polymyositis (PM), osteoarthritis (OA) of hip or knee, and ankylosing spondylitis (AS) with BC in European populations and East Asian populations using Mendelian randomized (MR) analysis.

**Methods:**

The genetic instruments linked to MSCTD, RA, SS, SLE, SSc, DM, PM, OA, and AS were chosen from the EBI database of complete genome-wide association studies (GWAS) summary data and the FinnGen consortium. The associations of genetic variants with BC were extracted from the Breast Cancer Association Consortium (BCAC). Two Sample MR was performed using summary data from GWAS, principally using the inverse variant weighted (IVW) method. Heterogeneity, pleiotropy, and sensitivity analyses were performed to evaluate the robustness of the results by weighted median, MR Egger, simple mode, weighted mode, and leave-one-out analysis.

**Results:**

In the European population, causal relationships between RA and BC (OR=1.04, 95%CI: 1.01-1.07, *P*=0.023), AS and BC (OR=1.21, 95%CI: 1.06-1.36, *P*=0.013) were confirmed. IVW analysis showed DM (OR=0.98, 95%CI: 0.96-0.99, *P*=0.026) and PM (OR=0.98, 95%CI: 0.97-0.99, *P*=0.002) were associated with slightly decreased risks of estrogen receptor (ER)+ BC, and MSCTD was associated with an increased risk of ER- BC (OR=1.85, 95%CI: 1.27-2.44, *P*=0.039). There was no causal relationship between SLE, SS, SSc, OA, and BC, neither ER+ BC nor ER- BC. However, in the East Asian population, IVW analysis showed that RA (OR=0.94, 95%CI: 0.89-0.99, *P*=0.0096) and SLE (OR=0.95, 95%CI: 0.92-0.99, *P*=0.0058) was associated with decreased risks of BC.

**Conclusions:**

This study suggests that causal relationships between patients with MSCTD and BC in the European population are different from those in the East Asian population, patients with RA and AS in the European population have an increased risk of BC, patients with MSCTD have increased risk of ER- BC in the European population, while patients with RA and SLE in the East Asian population have decreased risk of BC.

## Introduction

Diseases of the musculoskeletal system and connective tissue (MSCTD) including rheumatoid arthritis (RA), Sjogren syndrome (SS), systemic lupus erythematosus (SLE), systemic sclerosis (SSc), dermatomyositis (DM), polymyositis (PM), osteoarthritis (OA), and ankylosing spondylitis (AS) are a series of chronic inflammatory disease often caused by autoimmune imbalance. Because of the immune pathways underlying its pathogenesis, the risk of malignancies among MSCTD patients are of considerable interest. Although inflammatory myopathies were most considered for increased risks of malignancy, a wide spectrum of MSCTD had associations with particular types of cancer (1–3). The strength of association and types of cancer varied considerably by disease type (4). Women are more susceptible to most MSCDT due to estrogen which was also a major shared risk for breast cancer (BC). BC is the most widespread malignancy worldwide, with more than one million diagnoses every year. It is the most frequent cause of cancer in women and the second leading cause of cancer-related deaths in women in the United States, so many investigators also focused on rheumatic diseases (5). Epidemiological studies found that patients with RA had decreased risks of BC (6). Patients with SLE also had a lower risk of BC compared to that expected in the general population (7, 8). But a retrospective nationwide cohort study using databases from the National Health Insurance Service in Korea found breast and reproductive system (OR=1.5, 95% CI:1.2-1.7) cancers were at increased risk in 17,854 SLE patients (9). No more reports about associations between any other MSCTD, SSc, DM, PM, OA, and BC especially estrogen receptor (ER)+ BC or ER- BC were investigated.

Mendelian randomization (MR) was a strategy for investigating causal relationships that could efficiently circumvent the aforementioned restrictions by employing genetic variants as exposure instrumental variables (IVs) and avoiding potential confounders because of the random assignment of genetic variants (10, 11). Therefore, using summary data from available genome-wide association studies (GWAS), the present study was to assess the causal effect of MSCTD, RA, SS, SLE, SSc, DM, PM, OA, and AS in the European population or East Asian population on BC including ER+ BC or ER- BC by multiple MR methods.

## Methods

### Study design and data sources

**Figure 1:**
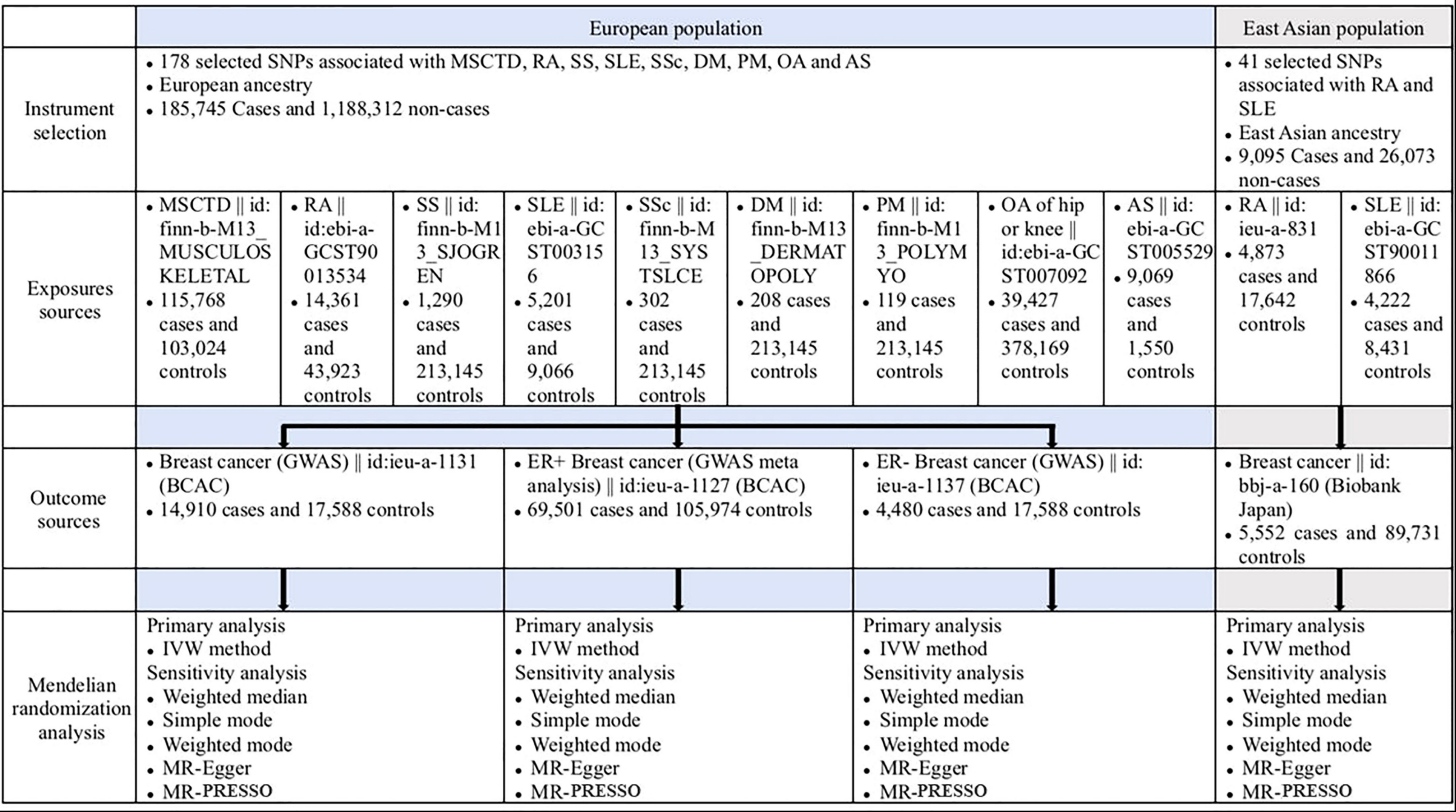
Overview of the study design (European population and East Asian population). AS, ankylosing spondylitis; BCAC, Breast Cancer Association Consortium; DM, dermatomyositis; IVW, inverse variance weighted; MR-PRESSO, Mendelian randomization pleiotropy residual sum and outlier; MSCTD, diseases of the musculoskeletal system and connective tissue; OA, osteoarthritis; PM, polymyositis; RA, rheumatoid arthritis; SLE, systemic lupus erythematosus; SNP, singlenucleotide polymorphism; SSc, systemic sclerosis; SS, Sjogren syndrome.

An overview of the study design is presented in **Figure 1**. The present study is based on data on MSCTD, RA, SS, SLE, SSc, DM, PM, OA, AS, and BC from the FinnGen study (12), Biobank Japan (13) and GWAS on BC (14). All studies had been approved by a relevant ethical review board and participants had given informed consent.

Genetic instrumental variables (IVs) for the exposures (178 selected SNPs associated with MSCTD, RA, SS, SLE, SSc, DM, PM, OA of hip and knee, and AS) in people of European ancestry were downloaded from the GWAS summary data (https://gwas.mrcieu.ac.uk/) and obtained from the EBI database of complete GWAS summary data and the FinnGen consortium (185,745 cases and 1,188,312 controls). Genetic IVs for the exposures (41 selected SNPs associated with RA and SLE) in people of East Asian ancestry were obtained from the EBI database of complete GWAS summary data and the IEU OpenGWAS consortium (9,095 cases and 26,073 controls). Study outcomes including BC (14,910 cases and 17,588 controls), ER+ BC (69,501 cases and 105,974 controls), and ER- BC (4,480 cases and 17,588 controls) were from the Breast Cancer Association Consortium (BCAC) in the European population. Study outcomes (BC) in people of East Asian ancestry were obtained from the Biobank Japan (5,552 cases and 89,731 controls).

### Genetic IVs selection

Single-nucleotide polymorphisms (SNPs) associated with MSCTD, RA, SS, SLE, SSc, DM, PM, OA of the hip and knee, and AS in the European and East Asian populations at the genome-wide significance level (*P*<5×10^-8^) were separately derived from a GWAS with a total of 1,374,057 European individuals (185,745 cases and 1,188,312 controls) and 35,168 East Asian individuals (9,095 cases and 26,073 controls). After the removal of SNPs with linkage disequilibrium (*r* ^2^≥0.01) and SNPs with minor allele frequency (MAF) of less than 0.3, 178, and 41 SNPs were selected as IVs in the analysis in the European populations (**Supplementary Table 1**) and East Asian populations (**Supplementary Table 2**), respectively. Odds ratios (ORs) and corresponding confidence intervals (CIs) of the associations were scaled in log-transformed odds of genetic liability to MSCTD in the main analysis.

*F* statistics for each SNP solely were calculated by the following equation: F=R^2^(N - 2)/(1 -R^2^). R^2^ represented the variance of each collected IV on MSCTD. N was the sample size of the original GWAS research (15). To calculate R^2^ for each IV, we used the following formula: R^2 =^ 2β^2^EAF(1-EAF)/2β^2^EAF(1-EAF) + (se(β))^2^NEAF(1-EAF) where EAF was the effect allele frequency, the beta was the estimated genetic effect on physical activity, N was the sample size of the GWAS and se was the standard error of the genetic effect (16). IVs with F statistics of less than ten were considered weak instruments and would be excluded from MR analysis (15).

### Statistical analysis

The inverse variance weighted (IVW) method was used for the primary MR analyses. The weighted median, MR-Egger regression (17), and Mendelian randomization pleiotropy residual sum and outlier (MR-PRESSO) methods were performed as sensitivity analyses. Simple mode and weighted mode were also conducted to test the robustness of the results (18).

The intercept test of MR-Egger regression was used to identify horizontal pleiotropy (19). MR-PRESSO method can detect outliers of SNPs with pleiotropic effects and provide estimates after the removal of outliers (20). Heterogeneity among estimates of SNPs was quantified by Cochran’s *Q* value. The asymmetry of the funnel plot can also be considered an indicator of horizontal pleiotropy (21). A leave-one-out (LOO) analysis was performed to assess whether a single SNP drive the association and random-effects modes IVW was used to find any change in the results. The scatter and Funnel plots from the MR analysis are used to visually compare this part of the results. All statistical analyses were conducted using the Two-Sample MR package in R statistical software (version 4.2.1) and a *P* value <0.05 was regarded as statistically significant.

## Results

### MSCTD and BC in the European population

**Figure 2:**
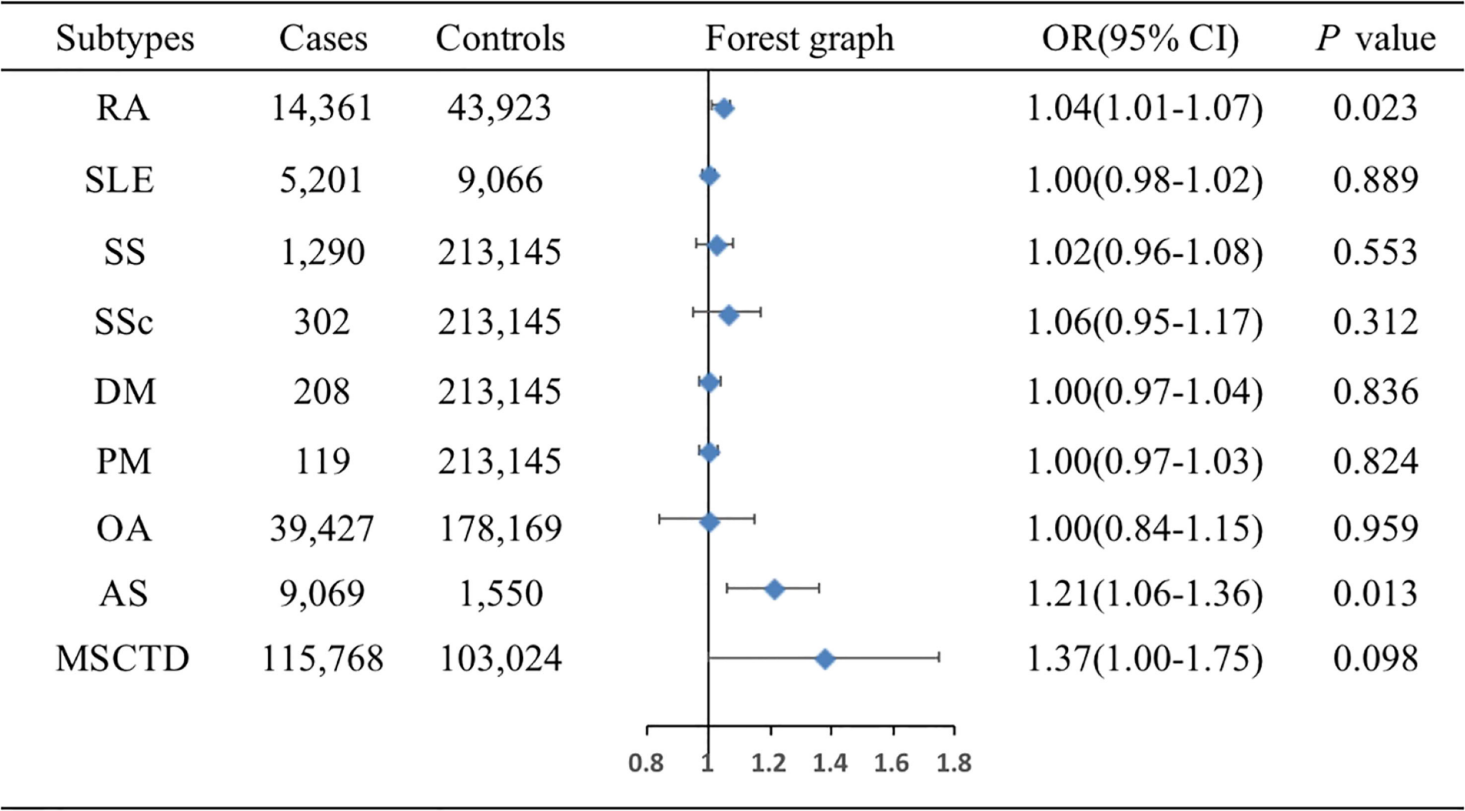
Associations of genetic liability to MSCTD with risk of BC in the European individuals. AS, ankylosing spondylitis; BC, breast cancer; DM, dermatomyositis; MSCTD, diseases of the musculoskeletal system and connective tissue; OA, osteoarthritis; PM, polymyositis; RA, rheumatoid arthritis; SLE, systemic lupus erythematosus; SSc, systemic sclerosis; SS, Sjogren syndrome; CI, confidence interval; OR, odds ratio.

The associations of genetic liability to MSCTD, RA, SS, SLE, SSc, DM, PM, OA of hip and knee, and AS with BC were displayed in **Figure 2**. In the European population, genetic predisposition to RA (IVW method) showed significant association with a slightly increased risk of BC (OR=1.04, 95%CI: 1.01-1.07, *P*=0.023), although MR-Egger (*P*=0.086) did not duplicate the result of IVW. We detected high heterogeneity (Cochran’s *Q* value=112.276, *P*=0.015) but no horizontal pleiotropy (*P*=0.712) in the analysis (**Supplementary Table 3**). IVW analysis also showed a causal relationship between AS and BC (OR=1.21, 95%CI: 1.06-1.36, *P*=0.013) without heterogeneity (*P*=0.708) and horizontal pleiotropy (*P*=0.201) (**Supplementary Table 4**). Neither the IVW analysis nor the MR-Egger regression, the simple mode, the weighted median approaches showed MSCTD (OR=1.37, 95%CI: 1.00-1.57, *P*=0.098), SS (OR=1.02, 95%CI: 0.96-1.08, *P*=0.553), SLE (OR=1.00, 95%CI: 0.98-1.02, *P*=0.889), SSc (OR=1.06, 95%CI: 0.95-1.17, *P*=0.312), DM (OR=1.00, 95%CI: 0.97-1.04, *P*=0.836), PM (OR=1.00, 95%CI: 0.97-1.03, *P*=0.824), or OA of hip and knee (OR=1.00, 95%CI: 0.84-1.15, *P*=0.959) to be linked with BC (**Figure 2**). There were no heterogeneity and pleiotropy among these SNPs in the sensitivity analysis.

**Figure 3:**
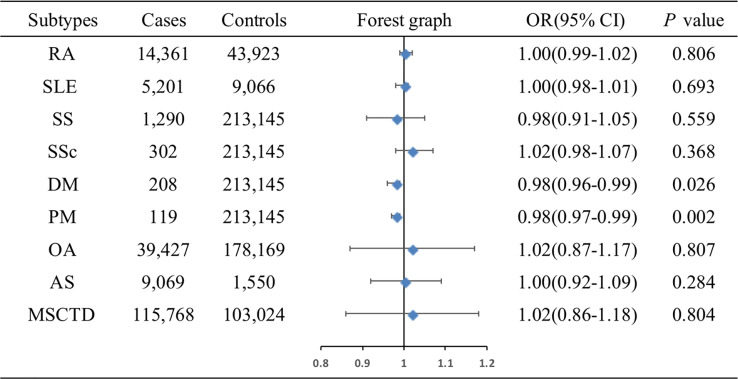
Associations of genetic liability to MSCTD with risk of ER+ BC in the European individuals. AS, ankylosing spondylitis; BC, breast cancer; CI, confidence interval; ER, estrogen receptor, DM, dermatomyositis; MSCTD, diseases of the musculoskeletal system and connective tissue; OA, osteoarthritis; OR, odds ratio; PM, polymyositis; RA, rheumatoid arthritis; SLE, systemic lupus erythematosus; SSc, systemic sclerosis; SS, Sjogren syndrome.

The associations of genetic liability to MSCTD, RA, SS, SLE, SSc, DM, PM, OA of hip and knee, and AS with ER+ BC were shown in **Figure 3**. MR analysis showed DM (OR=0.98, 95%CI: 0.96-0.99, *P*=0.026) and PM (OR=0.98, 95%CI: 0.97-0.99, *P*=0.002) were associated with slightly decreased risks of ER+ BC by Wald ratio because only 1 SNP was selected for DM or PM. IVW analysis showed MSCTD (OR=1.02, 95%CI: 0.86-1.18, *P*=0.804), RA (OR=1.00, 95%CI: 0.99-1.02, *P*=0.806), SLE (OR=1.00, 95%CI: 0.98-1.01, *P*=0.693), SS (OR=0.98, 95%CI: 0.91-1.05, *P*=0.559), SSc (OR=1.02, 95%CI: 0.98-1.07, *P*=0.368), OA of hip and knee (OR=1.02, 95%CI: 0.87-1.17, *P*=0.807), and AS (OR=1.00, 95%CI: 0.92-1.09, *P*=0.284) were not associated with ER+ BC (**Figure 3**). There were no heterogeneity and pleiotropy among these SNPs in the sensitivity analysis.

**Figure 4:**
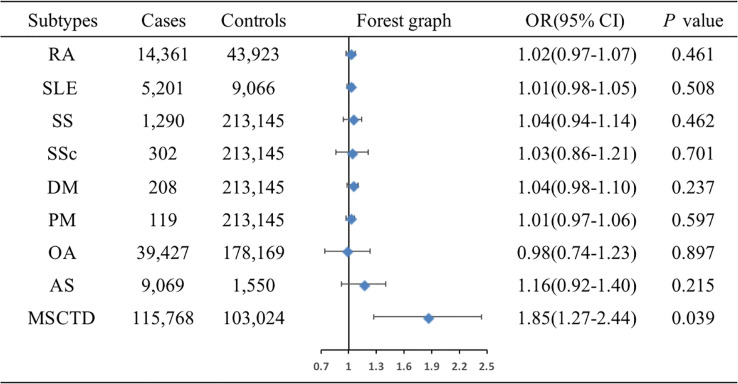
Associations of genetic liability to MSCTD with risk of ER- BC in the European individuals. AS, ankylosing spondylitis; BC, breast cancer; CI, confidence interval; ER, estrogen receptor, DM, dermatomyositis; MSCTD, diseases of the musculoskeletal system and connective tissue; OA, osteoarthritis; OR, odds ratio; PM, polymyositis; RA, rheumatoid arthritis; SLE, systemic lupus erythematosus; SSc, systemic sclerosis; SS, Sjogren syndrome.

The associations of genetic liability to MSCTD, RA, SS, SLE, SSc, DM, PM, OA of hip and knee, and AS with ER- BC were shown in **Figure 4**. IVW analysis showed only MSCTD was associated with an increased risk of ER- BC (OR=1.85, 95%CI: 1.27-2.44, *P*=0.039) (**Supplementary Table 5**). There were no causal relationships between RA, SS, SLE, SSc, DM, PM, OA of hip and knee, AS and ER- BC.

### MSCTD and BC in the East Asian population

**Figure 5:**
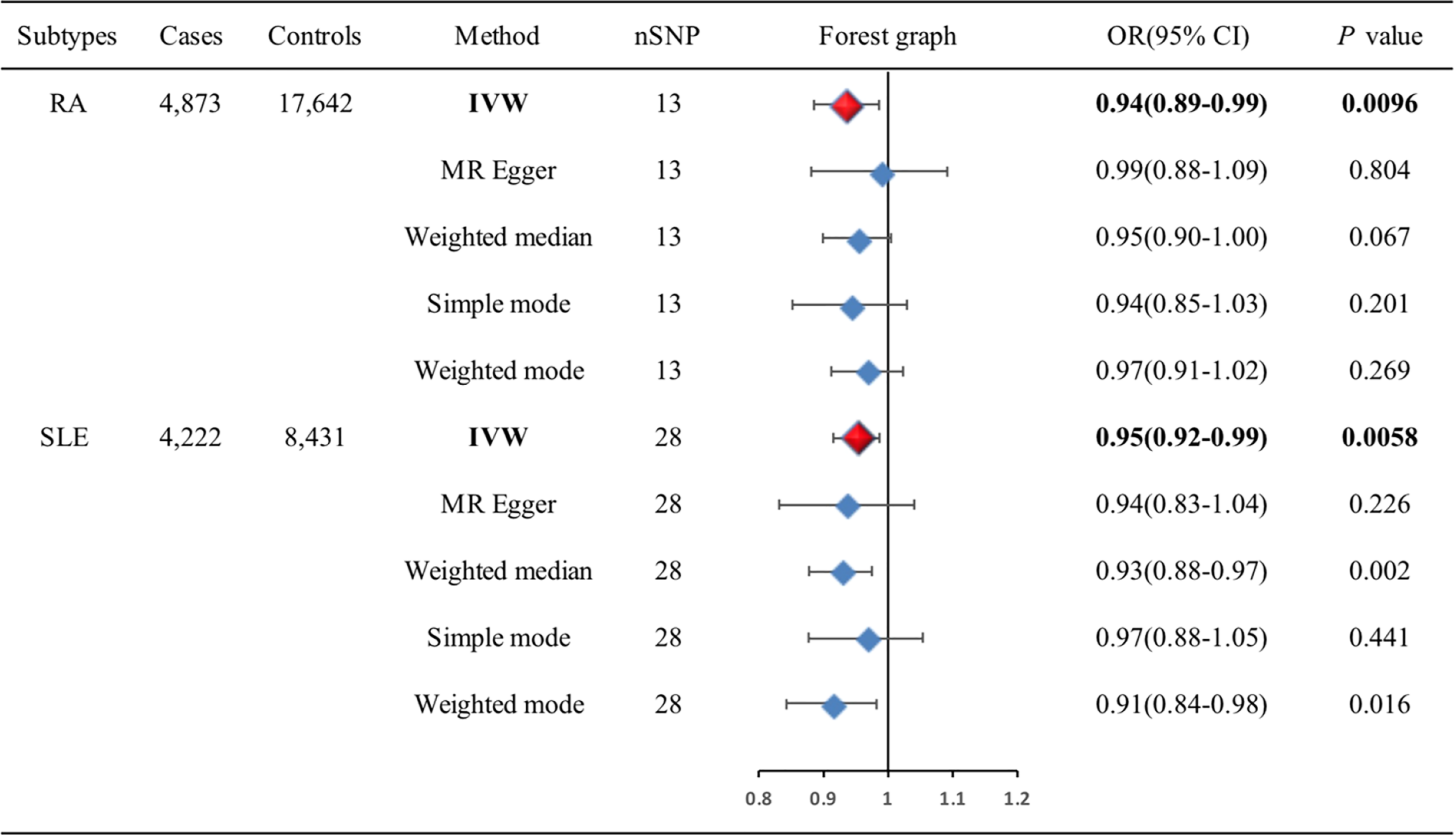
Associations of genetic liability to RA and SLE with risk of BC in the East Asian individuals. BC, breast cancer; CI, confidence interval; IVW, inverse variance weighted; OR, odds ratio; RA, rheumatoid arthritis; SLE, systemic lupus erythematosus; SNP, single-nucleotide polymorphism.

**Figure 6:**
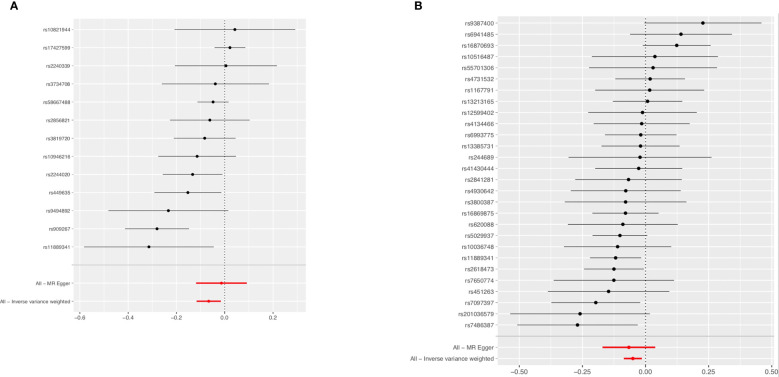
Forest plot of the effects of RA **(A)** and SLE **(B)** on BC in the East Asian population. BC, breast cancer; RA, rheumatoid arthritis; SLE, systemic lupus erythematosus.

The associations of genetic liability to RA and SLE with BC were shown in **Figure 5**. However, in the East Asian population, genetic predisposition to RA (IVW method) showed a significant association with a slightly decreased risk of BC (OR=0.94, 95%CI: 0.89-0.99, *P*=0.0096) (**Figure 6A**), although MR-Egger (*P*=0.804) did not duplicate the result of IVW. We detected high heterogeneity (Cochran’s *Q* value=26.893, *P*=0.008) but no horizontal pleiotropy (*P*=0.288) in the analysis (**Supplementary Table 6**).

IVW analysis showed SLE (OR=0.95, 95%CI: 0.92-0.99, *P*=0.0058) was associated with a slightly decreased risks of BC (**Figure 6B**). Weighted median (OR=0.93, 95%CI: 0.88-0.97, *P*=0.002) and weighted mode (OR=0.91, 95%CI: 0.84-0.98, *P*=0.016) methods yielded similar results. There was no heterogeneity and pleiotropy among these SNPs in the sensitivity analysis (**Supplementary Table 7**).

## Discussion

In the current MR study, in the European population, we found that genetic liability to RA was associated with a slightly increased risk of BC, and genetic liability to AS was associated with an obviously increased risk of BC. Genetic liabilities to DM and PM were associated with a slightly decreased risk of ER+ BC, but results needed further investigation because only one SNP was selected for either DM or PM. Genetic liability to MSCTD was associated with a significantly increased risk of ER- BC. There were no causal relationships between SLE, SS, SSc, OA, and BC, neither ER+ BC nor ER- BC. However, in the East Asian population, genetic liabilities to RA and SLE were associated with a slightly decreased risk of BC.

Many researchers have focused on the association between BC and RA rather than other rheumatic diseases; most previous observational studies had found the risk of BC in RA patients was decreased, but still varied in different populations and different databases (22–29). Early in 2008, the EMECAR (Estudio de la Morbilidad y Expresioín Cliínica de la Artritis Reumatoide) cohort study including 789 randomly selected RA patients (1999-2005) from 34 centers in Spain found, compared with the expected rate, RA in Spanish women did not have an increased risk of developing BC with an incidence rate (IR) of 13 (3 to 49) per 10,000 and standardized incidence ratio (SIR) of 0.9 (0.1-3.2) (24). Two meta-analyses, one being an update of the other, including 23 studies published from 1990 to 2014 compared malignancy risk in RA patients with the general population. The analysis (1990-2007) revealed the risk of BC in RA was decreased, compared with the general population [9 studies, SIR: 0.84 (0.79-0.90)], but an updated meta-analysis (1990-2014) found there was no increased or decreased risk of BC in RA [19 studies, SIR: 0.86 (0.73-1.01)] (6, 30). Another meta-analysis in 2014 did not find any risk of BC in RA patients (SIR=0.86, 95% CI=0.72-1.02), either. However, results of the analysis in the subgroup showed that BC risk in RA patients was different, decreased in Caucasians (SIR:0.82, 95% CI:0.73-0.93) but increased non-Caucasians (SIR:1.21, 95% CI:1.19-1.23) (31). A large population-based study including 15,921 Swedish women with RA and 79,441 matched population referents from 2006 to 2016 found the risk of incident BC in women with RA was reduced and the association was not attenuated by adjustment for BC risk factors (HR=0.80, 95% CI: 0.68-0.93) (32). In our MR analysis, BC risk in RA patients was also different between the European population and the East Asian population, which confirmed the results from the meta-analysis. But RA in the European population had a slightly increased risk of BC (OR=1.04, 95%CI: 1.01-1.07, *P*=0.023)) and slightly decreased risk of BC in the East Asian population (OR=0.94, 95%CI: 0.89-0.99, *P*=0.0096) which were still inconsistent with previous study.

Some causes might explain the inconclusive results of the causal association between RA and BC. First, the results of the meta-analysis in 2014 and our MR analysis showed the ethnic differences in the association might be due to the variation of genetic predisposition, SNPs in the *DRB1* gene which were primary genetic susceptibility locus for RA, and various between different races (33), had recently been linked to a decreased risk of BC (34). The inconsistency of results might also be due to different databases. Second, the correlation of some ERα genotypes with BC and some autoimmune diseases including RA had been previously reported (35, 36). In the different populations, various lifestyles and environmental factors resulted in different gene-environment interactions and thus might also explain different cancer susceptibilities. Third, it seemed that the decreased age- and sex-specific incidence might be potential factors to decrease the risk of BC in RA. Younger RA (diagnosed before 50 years old) was a risk factor for BC with higher SIR (25), SIR in females with age < 40 was even up to 2.19. However, the risk tended to be decreased with aging (23). However, the risk of BC was increased in the European population with RA while decreased in the East Asian population in our MR analysis, there were no causal relationships between RA and ER+ BC or ER- BC in the European population. Although BC risk prediction remained unsatisfied, this might provide preference for the risk of BC in different populations.

SLE patients seemed to have decreased risk for certain hormone-sensitive cancers including BC in a previous study. A multisite international SLE cohort (30 centers, 16,409 patients with SLE) in Canada was conducted to observe the risk of cancers. A decreased risk was estimated for BC (SIR=0.73, 95% CI: 0.61-0.88) in SLE, compared with the general population (7). Conversely, a retrospective nationwide cohort study including 17,854 SLE patients using databases from the National Health Insurance Service in Korea found that BC and reproductive system cancers (SIR=1.50, 95% CI: 1.20-1.70) occurred predominantly in SLE patients, compared to the general population (9). In a recent meta-analysis including 47,325 SLE patients, decreased risk for BC (376 BC cases) in SLE was shown (SIR=0.76, 95% CI:0.69-0.85) (37). We found in most cohorts of this meta-analysis, the patients were primarily Caucasian, whereas the study of Kang et al. featured Asian patients. This suggested that perhaps the cancer profile of SLE patients might be influenced by race, but BC risk was not stratified by race in this meta-analysis. In our MR analysis, genetic liabilities to SLE were associated with a slightly decreased risk of BC in the East Asian population which was consistent with the result of the meta-analysis. But there were no causal relationships between SLE and BC, neither ER+ BC nor ER- BC.

Epidemiological studies had described and confirmed the link between DM or PM and BC. In a recent meta-analysis, patients with DM (SIR=5.50, 95% CI: 4.31-6.70) and PM (SIR=1.62, 95% CI: 1.19-2.04) were more likely to develop malignancies than the general population (38). An increased risk was estimated for BC in DM (SIR=3.49, 95% CI:3.26-3.72), and an increased risk was also estimated for BC in PM (SIR=1.67, 95% CI:1.48-1.86) (38). Compared with other rheumatic diseases, PM and DM (especially DM) were significantly associated with the risks of a larger number of malignancies, including lung, kidney, breast, bladder, endometrial, cervical and thyroid cancers, lymphoma, myeloma, and brain tumor for PM, and lung, ovarian, breast, colorectal, cervical, bladder, nasopharyngeal, esophageal, pancreatic, colon, and kidney cancers for DM. These results implied that PM and DM were similar but different diseases regarding the types of associated malignancies. Hendren H also identified 44 previously published cases of DM in patients with BC, 22 patients had specific staging and a confirmed DM diagnosis, demonstrating BC patients also presented with proximal muscle weakness, skin rashes, or other systemic manifestations suggestive of DM (39). In our MR analysis, we did not find a causal relationship between DM or PM and BC, too small a sample size of cases (only 208 cases for DM and 119 cases for PM) might be the main reason for these negative results. Although genetic liabilities to DM and PM were associated with a slightly decreased risk of ER+ BC, results needed further investigation in larger samples of cases because only one SNP was selected for either DM or PM.

The association between SSc and BC was contradictory and inconclusive in previous research (40–42). In a population-based cohort of 441 patients with scleroderma in South Australia, 8 cases of BC were identified, SIR of BC in female SSc patients was found to be 1.62 (95% CI: 0.70-3.19) (43). In another population-based cohort of 318 patients with scleroderma in Italy, 12 cases of BC were recorded, SIR of BC in SSc patients was 2.10 (95% CI: 1.13-3.90), compared with the general population (44). Among 33 cases of SSc with BC from an observational retrospective multicenter study in northern Italy, 75% of BC were ER+ and 25% of BC were ER- (45). In our MR analysis, there was no causal relationship between SSc and BC, neither ER+ BC nor ER- BC, which was the same as most of the previous studies. Too small a sample of SSc cases (only 302 cases) might also be the main reason for negative results, which needed further investigation in larger samples of cases.

In our MR analysis, genetic liability to AS was associated with an obviously increased risk of BC in the European population, but AS was not associated with the risk of ER+ BC or ER- BC. Association between AS and BC was seldom studied except for any small samples of case reports. In 2015, 31 cases of AS with malignancies accounted for 11.8% of all AS patients admitted to Beijing University First Hospital in the same period were reported. Among the 31 malignancies with AS patients, only 2 cases with BC were reported (46).

In our MR analysis, MSCTD was associated with an increased risk of ER- BC in the European population. There was no causal relationship between SS, OA, and BC, neither ER+ BC nor ER- BC in the European population. All these associations between the risk of BC and MSCTD were seldom reported before.

An important strength of this MR analysis was the data from different populations, which diminished confounding and reverse causality as well as examined the ancestral difference in the associations. We performed MR analysis in the European and East Asian populations, separately, which minimized bias caused by population structure bias.

A major limitation of the current MR study was that we did not obtain any more data about SSc, SS, DM, PM, OA, AS, and MSCTD in the East Aisan population. And in the European population, SSc, DM, and PM had relatively small numbers of cases, which resulted in low precision in MR estimation and possible false negative findings. Another disadvantage was that we did not separate the association between BC and rheumatic diseases from the association reported for several medications, it was important to avoid bias introduced by different patterns of drug use. Increased risks of malignancy were well documented in patients treated with cyclophosphamide (47), mycophenolate (48), azathioprine (49), methotrexate (50), and biologic agents (51). Previous studies have shown that immunosuppression is related to tumorigenesis, and may affect tumor recurrence when using DMARDs. MTX was associated with a two-fold increased risk of skin cancer, but low doses of MTX do not increase the risk of cancers other than skin cancer. Lymphomas and non-melanoma skin cancers may be associated with Anti-TNF agents as well, results from the ARTIS program (52). Fortunately, a study published this year found that it was unclear whether long-term use of DMARDs increased the risk of malignancies. Such as MTX, Anti-TNF agents, or other biologics, did not induce tumor or hematological malignancies (53).

In conclusion, this study suggests that in the European population, RA and AS may play a role in the development of BC and MSCTD may play a role in the development of ER- BC. However, in the East Asian population, RA and SLE are associated with a slightly decreased risk of BC. Future studies are needed to verify our findings.

## Data availability statement

The original contributions presented in the study are included in the article/**Supplementary Material**. Further inquiries can be directed to the corresponding author.

## Ethics statement

Ethical approval was waived, and informed consent of participants was obtained previously due to the published GWAS summary statistics.

## Author contributions

All authors have participated in the study and have read and approved the manuscript, agreed to be accountable for the content of this work. Y-CX: Data curation, Methodology, Software, Writing-Original draft preparation, Investigation. J-XW, Y-RC, HQ: Data curation, Software. H-YW: Methodology, statistical analysis. FW: Conceptualization, Writing - review & editing, Project administration, Funding acquisition. All authors contributed to the article and approved the submitted version.
