# Corrigendum: Diseases of the musculoskeletal system and connective tissue and risk of breast cancer: mendelian randomization study in European and East Asian populations

**DOI:** 10.3389/fonc.2023.1254132

**Published:** 2023-07-28

**Authors:** Yue-chen Xu, Jian-xiong Wang, Yi-ran Chu, Han Qian, Hong-yan Wang, Fan Wang

**Affiliations:** ^1^Department of Radiotherapy, The First Affiliated Hospital of Anhui Medical University, Hefei, China; ^2^Department of Rheumatology and Immunology, The First Affiliated Hospital of Anhui Medical University, Hefei, China

**Keywords:** connective tissue disease, breast cancer, European, East Asian, Mendelian randomization

In the published article, there was an error in the author list, and author Sheng-qian Xu was erroneously included. The corrected author list appears below.

Yue-chen Xu1†, Jian-xiong Wang2†, Yi-ran Chu2, Han Qian1, Hong-yan Wang1, Fan Wang1*

Consequently, the Author Contributions section erroneously listed the author Sheng-qian Xu. The correct Author Contributions appears below:

**Author contributions**

All authors have participated in the study and have read and approved the manuscript, agreed to be accountable for the content of this work. Y-CX: Data curation, Methodology, Software, Writing-Original draft preparation, Investigation. J-XW, Y-RC, HQ: Data curation, Software. H-YW: Methodology, statistical analysis, FW: Conceptualization, Writing - review & editing, Project administration, Funding acquisition.

The authors apologize for this error and state that this does not change the scientific conclusions of the article in any way. The original article has been updated.

