# Supplementary material for: Diseases of the musculoskeletal system and connective tissue and risk of breast cancer: Mendelian randomization study in European and East Asian populations
Source: Front Oncol. 2023 Apr 26;13:1170119. doi: 10.3389/fonc.2023.1170119 (PMC10169740; doi:10.3389/fonc.2023.1170119)

**Two sample MR report**

**Rheumatoid arthritis || id:ebi-a-GCST90013534 against Breast cancer (GWAS) || id:ieu-a-1131**

Date: **29 January, 2023**

**Results from two sample MR:**

| **method** | **nsnp** | **b** | **se** | **pval** |
| --- | --- | --- | --- | --- |
| MR Egger | 83 | 0.0467231 | 0.0268846 | 0.0860262 |
| Weighted median | 83 | 0.0302856 | 0.0243919 | 0.2143742 |
| Inverse variance weighted | 83 | 0.0390926 | 0.0171594 | 0.0227140 |
| Simple mode | 83 | 0.0340333 | 0.0546853 | 0.5354397 |
| Weighted mode | 83 | 0.0375000 | 0.0228592 | 0.1047361 |

**Heterogeneity tests**

| **method** | **Q** | **Q_df** | **Q_pval** |
| --- | --- | --- | --- |
| MR Egger | 112.0869 | 81 | 0.0126651 |
| Inverse variance weighted | 112.2764 | 82 | 0.0148549 |

**Test for directional horizontal pleiotropy**

| **egger_intercept** | **se** | **pval** |
| --- | --- | --- |
| -0.0016104 | 0.004352 | 0.7123164 |

**Test that the exposure is upstream of the outcome**

| **snp_r2.exposure** | **snp_r2.outcome** | **correct_causal_direction** | **steiger_pval** |
| --- | --- | --- | --- |
| 0.1419846 | 0.0036726 | TRUE | 0 |

Note - R^2^ values are approximate

Calculated as F=N-κ-1/κ × R^2^/1-R^2^

| \| SNP \| b \| se \| p \| SNP \| b \| se \| p \| \| --- \| --- \| --- \| --- \| --- \| --- \| --- \| --- \| \| rs10435844 \| 0.409439 \| 0.232143 \| 0.077776 \| rs3025669 \| -0.34807 \| 0.462904 \| 0.4521 \| \| rs10911902 \| 0.391972 \| 0.286895 \| 0.171859 \| rs3087243 \| -0.20936 \| 0.140365 \| 0.135824 \| \| rs11123811 \| 0.099497 \| 0.175879 \| 0.571588 \| rs3134883 \| -0.15237 \| 0.189707 \| 0.421864 \| \| rs112733823 \| 0.041361 \| 0.135602 \| 0.760351 \| rs34046593 \| 0.04571 \| 0.133615 \| 0.732272 \| \| rs114508013 \| -0.27316 \| 0.115369 \| 0.0179 \| rs34502849 \| 0.008226 \| 0.211516 \| 0.968979 \| \| rs115521560 \| 0.080604 \| 0.055471 \| 0.146199 \| rs3757387 \| -0.13026 \| 0.156958 \| 0.406597 \| \| rs11574914 \| 0.084996 \| 0.161318 \| 0.598276 \| rs3761959 \| -0.02016 \| 0.236559 \| 0.932081 \| \| rs117026326 \| -0.45066 \| 0.489501 \| 0.357237 \| rs3806624 \| 0.095017 \| 0.20394 \| 0.641281 \| \| rs11754264 \| -0.0206 \| 0.347314 \| 0.952696 \| rs403214 \| 0.358862 \| 0.206783 \| 0.082661 \| \| rs11889341 \| 0.078445 \| 0.143929 \| 0.585737 \| rs42034 \| 0.509759 \| 0.231917 \| 0.027948 \| \| rs12126142 \| 0.376831 \| 0.23968 \| 0.115899 \| rs4409785 \| -0.15071 \| 0.238289 \| 0.527074 \| \| rs1234313 \| -0.06274 \| 0.23463 \| 0.789177 \| rs4602367 \| 0.045333 \| 0.232 \| 0.845078 \| \| rs12466919 \| -0.25073 \| 0.176585 \| 0.15564 \| rs4717901 \| -0.42088 \| 0.387149 \| 0.276976 \| \| rs12530098 \| 0.442836 \| 0.28437 \| 0.119411 \| rs4795400 \| 0.084791 \| 0.236878 \| 0.720377 \| \| rs12918327 \| 0.174164 \| 0.290657 \| 0.549035 \| rs5020946 \| 0.030373 \| 0.029299 \| 0.299899 \| \| rs13103285 \| 0.250758 \| 0.176946 \| 0.156441 \| rs502919 \| -0.40048 \| 0.268999 \| 0.136543 \| \| rs1355208 \| 0.55868 \| 0.222494 \| 0.012039 \| rs5754104 \| 0.276094 \| 0.250281 \| 0.269967 \| \| rs139395255 \| -0.1221 \| 0.174015 \| 0.482898 \| rs6011186 \| 0.268156 \| 0.361266 \| 0.457925 \| \| rs146305655 \| 0.196164 \| 0.107102 \| 0.067018 \| rs61828284 \| 0.242319 \| 0.195738 \| 0.215725 \| \| rs1538981 \| -0.43815 \| 0.260805 \| 0.092957 \| rs62422878 \| 0.024108 \| 0.215043 \| 0.910738 \| \| rs1571878 \| -0.03379 \| 0.11501 \| 0.768922 \| rs6421571 \| -0.17836 \| 0.178358 \| 0.317311 \| \| rs1595260 \| -0.26864 \| 0.230769 \| 0.244382 \| rs6479800 \| -0.1381 \| 0.174709 \| 0.42925 \| \| rs1611236 \| 0.157082 \| 0.160515 \| 0.327773 \| rs660442 \| 0.396439 \| 0.202437 \| 0.050191 \| \| rs1858037 \| -0.03953 \| 0.181818 \| 0.827903 \| rs6679677 \| 0.14687 \| 0.052284 \| 0.004969 \| \| rs1883832 \| -0.21673 \| 0.192015 \| 0.259019 \| rs7097397 \| -0.35655 \| 0.217237 \| 0.100734 \| \| rs1893592 \| 0.157787 \| 0.196721 \| 0.422505 \| rs7105899 \| -0.31117 \| 0.224592 \| 0.165907 \| \| rs1950897 \| -0.29747 \| 0.178672 \| 0.095928 \| rs71508903 \| -0.039 \| 0.150639 \| 0.79569 \| \| rs2069235 \| 0.00463 \| 0.150463 \| 0.975454 \| rs71565312 \| 0.354793 \| 0.134621 \| 0.008401 \| \| rs2073609 \| -0.27017 \| 0.581147 \| 0.642015 \| rs7170107 \| 0.016837 \| 0.144949 \| 0.907524 \| \| rs2076616 \| 0.126554 \| 0.213559 \| 0.553454 \| rs7206670 \| 0.286733 \| 0.261056 \| 0.272047 \| \| rs212389 \| -0.07467 \| 0.171078 \| 0.6625 \| rs740122 \| 0.787724 \| 0.268542 \| 0.003353 \| \| rs2233424 \| 0.114053 \| 0.231161 \| 0.621736 \| rs76153210 \| 0.12273 \| 0.392611 \| 0.754585 \| \| rs2258734 \| 0.307275 \| 0.206298 \| 0.136363 \| rs7731626 \| 0.026074 \| 0.099182 \| 0.792638 \| \| rs2275806 \| 0.153103 \| 0.246897 \| 0.535184 \| rs7749323 \| 0.004586 \| 0.19224 \| 0.98097 \| \| rs2301888 \| 0.333073 \| 0.143526 \| 0.020306 \| rs8032939 \| -0.01849 \| 0.163183 \| 0.909792 \| \| rs244685 \| -0.02472 \| 0.28427 \| 0.930706 \| rs8126756 \| -0.46659 \| 0.311057 \| 0.133614 \| \| rs28411352 \| 0.188184 \| 0.226477 \| 0.40602 \| rs9271365 \| 0.017799 \| 0.041326 \| 0.666692 \| \| rs2841275 \| -0.12678 \| 0.121212 \| 0.295599 \| rs9405192 \| 0.32809 \| 0.279775 \| 0.24092 \| \| rs28421442 \| 0.062399 \| 0.584279 \| 0.914951 \| rs9532434 \| -0.03421 \| 0.160526 \| 0.831237 \| \| rs2847297 \| 0.0299 \| 0.204873 \| 0.883964 \| rs9693589 \| -0.14818 \| 0.180124 \| 0.410701 \| \| rs2918392 \| 0.146707 \| 0.272455 \| 0.590258 \| rs9927316 \| -0.03091 \| 0.250552 \| 0.901832 \| \| rs9943599 \| -0.43373 \| 0.214458 \| 0.043128 \|  \|  \|  \|  \| |
| --- | --- | --- | --- | --- | --- | --- | --- | --- | --- | --- | --- | --- | --- | --- | --- | --- | --- | --- | --- | --- | --- | --- | --- | --- | --- | --- | --- | --- | --- | --- | --- | --- | --- | --- | --- | --- | --- | --- | --- | --- | --- | --- | --- | --- | --- | --- | --- | --- | --- | --- | --- | --- | --- | --- | --- | --- | --- | --- | --- | --- | --- | --- | --- | --- | --- | --- | --- | --- | --- | --- | --- | --- | --- | --- | --- | --- | --- | --- | --- | --- | --- | --- | --- | --- | --- | --- | --- | --- | --- | --- | --- | --- | --- | --- | --- | --- | --- | --- | --- | --- | --- | --- | --- | --- | --- | --- | --- | --- | --- | --- | --- | --- | --- | --- | --- | --- | --- | --- | --- | --- | --- | --- | --- | --- | --- | --- | --- | --- | --- | --- | --- | --- | --- | --- | --- | --- | --- | --- | --- | --- | --- | --- | --- | --- | --- | --- | --- | --- | --- | --- | --- | --- | --- | --- | --- | --- | --- | --- | --- | --- | --- | --- | --- | --- | --- | --- | --- | --- | --- | --- | --- | --- | --- | --- | --- | --- | --- | --- | --- | --- | --- | --- | --- | --- | --- | --- | --- | --- | --- | --- | --- | --- | --- | --- | --- | --- | --- | --- | --- | --- | --- | --- | --- | --- | --- | --- | --- | --- | --- | --- | --- | --- | --- | --- | --- | --- | --- | --- | --- | --- | --- | --- | --- | --- | --- | --- | --- | --- | --- | --- | --- | --- | --- | --- | --- | --- | --- | --- | --- | --- | --- | --- | --- | --- | --- | --- | --- | --- | --- | --- | --- | --- | --- | --- | --- | --- | --- | --- | --- | --- | --- | --- | --- | --- | --- | --- | --- | --- | --- | --- | --- | --- | --- | --- | --- | --- | --- | --- | --- | --- | --- | --- | --- | --- | --- | --- | --- | --- | --- | --- | --- | --- | --- | --- | --- | --- | --- | --- | --- | --- | --- | --- | --- | --- | --- | --- | --- | --- | --- | --- | --- | --- | --- | --- | --- | --- | --- | --- | --- | --- | --- | --- | --- | --- | --- | --- | --- | --- | --- | --- | --- | --- | --- | --- | --- | --- | --- | --- | --- | --- | --- | --- | --- | --- |


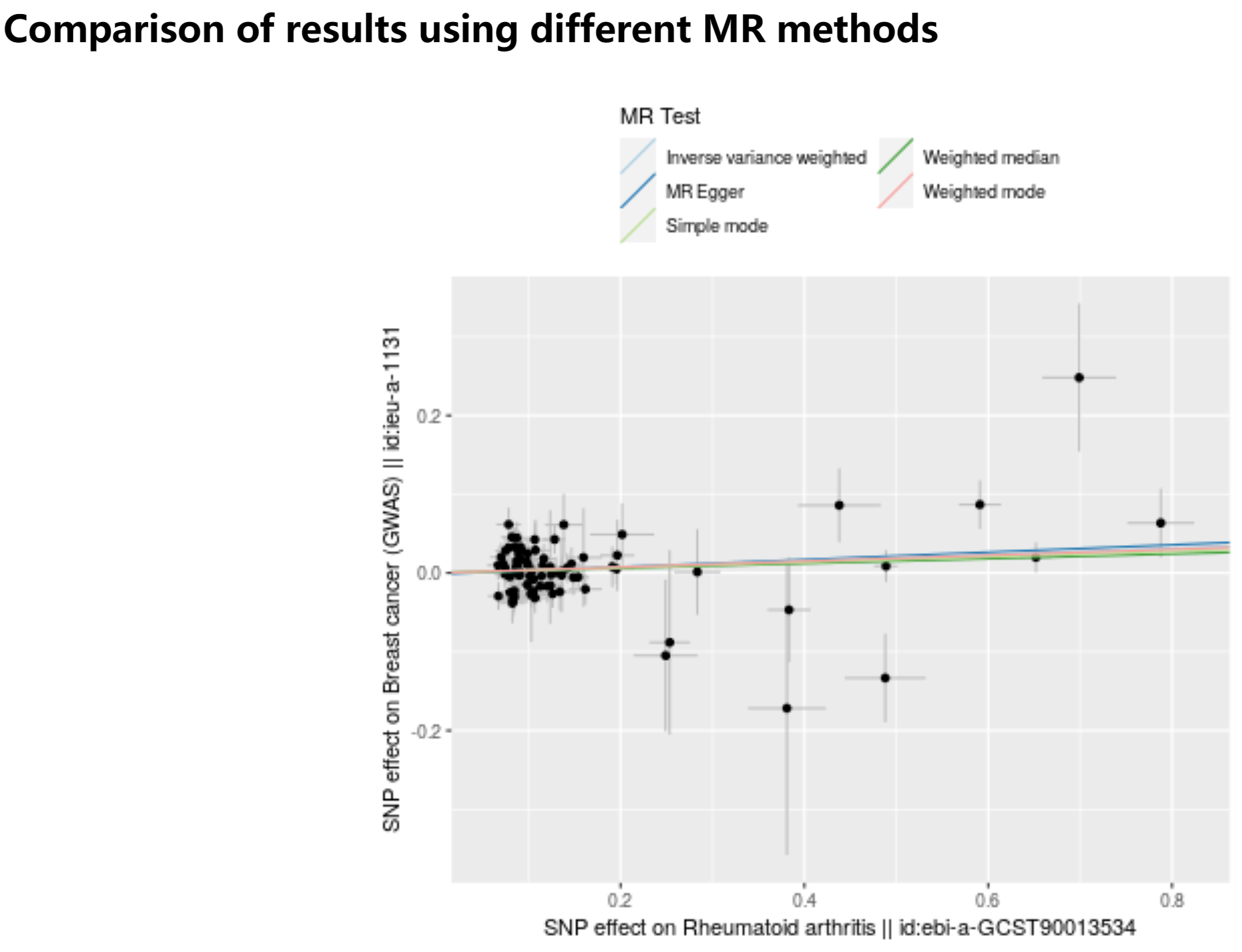

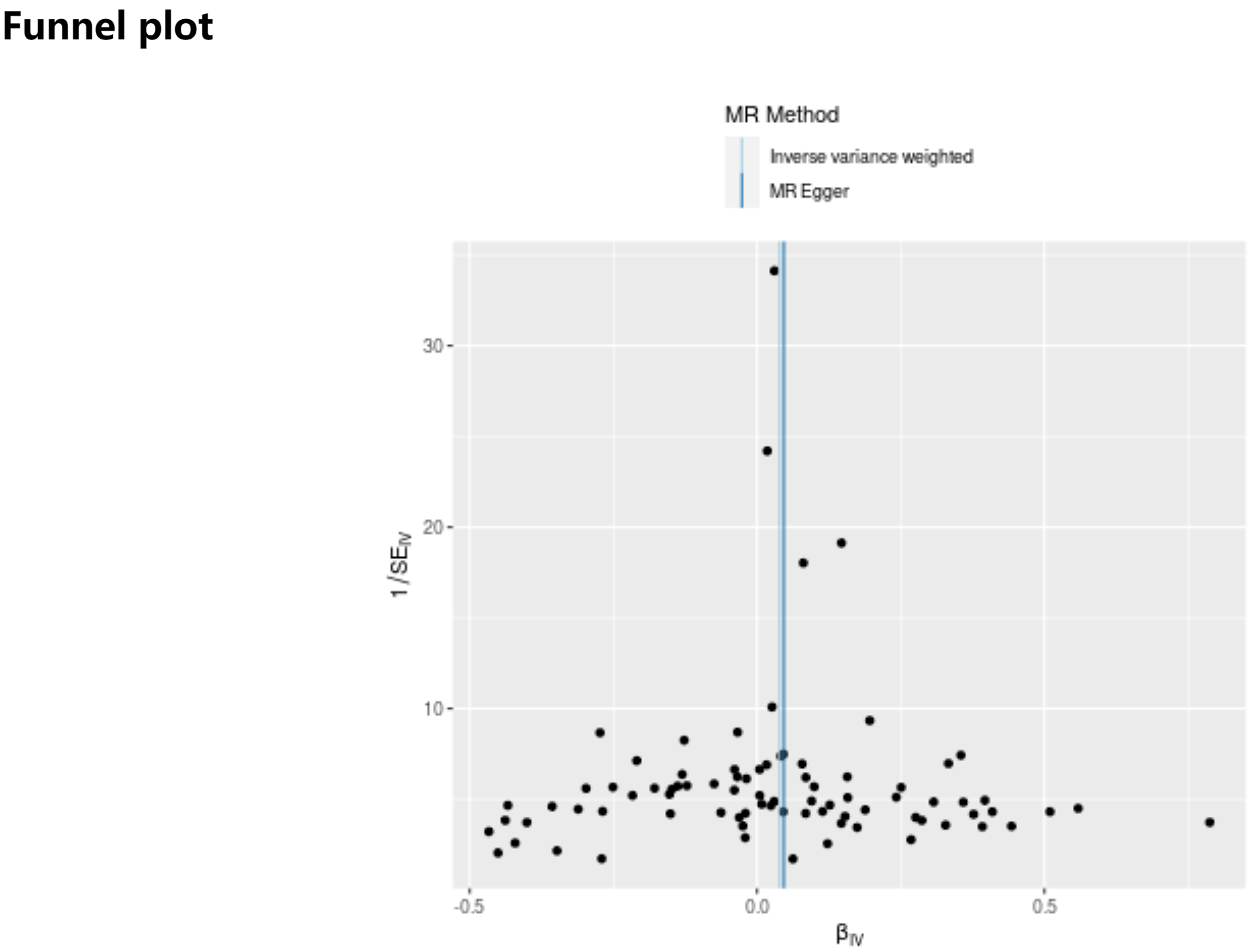

Supplement: Supplementary file 1 [file DataSheet_1.docx]
