# Supplementary material for: Diseases of the musculoskeletal system and connective tissue and risk of breast cancer: Mendelian randomization study in European and East Asian populations
Source: Front Oncol. 2023 Apr 26;13:1170119. doi: 10.3389/fonc.2023.1170119 (PMC10169740; doi:10.3389/fonc.2023.1170119)

**Two sample MR report**

**Ankylosing spondylitis || id:ebi-a-GCST005529 against Breast cancer (GWAS) || id:ieu-a-1131**

Date: **28 January, 2023**

**Results from two sample MR:**

| **method** | **nsnp** | **b** | **se** | **pval** |
| --- | --- | --- | --- | --- |
| MR Egger | 23 | 0.0539244 | 0.1304314 | 0.6834822 |
| Weighted median | 23 | 0.1882661 | 0.1048731 | 0.0726250 |
| Inverse variance weighted | 23 | 0.1925947 | 0.0772928 | 0.0127114 |
| Simple mode | 23 | 0.2065942 | 0.2077596 | 0.3308446 |
| Weighted mode | 23 | 0.1514060 | 0.1022917 | 0.1530152 |

**Heterogeneity tests**

| **method** | **Q** | **Q_df** | **Q_pval** |
| --- | --- | --- | --- |
| MR Egger | 16.22985 | 21 | 0.7566005 |
| Inverse variance weighted | 17.97194 | 22 | 0.7076507 |

**Test for directional horizontal pleiotropy**

| **egger_intercept** | **se** | **pval** |
| --- | --- | --- |
| 0.0091721 | 0.0069492 | 0.2010828 |

**Test that the exposure is upstream of the outcome**

| **snp_r2.exposure** | **snp_r2.outcome** | **correct_causal_direction** | **steiger_pval** |
| --- | --- | --- | --- |
| 0.3233897 | 0.0007473 | TRUE | 0 |

Note - R^2^ values are approximate

Calculated as F=N-κ-1/κ × R^2^/1-R^2^

| \| SNP \| b \| se \| p \| \| --- \| --- \| --- \| --- \| \| rs1041926 \| -0.63612 \| 0.816527 \| 0.43595 \| \| rs11190133 \| 0.593496 \| 0.561016 \| 0.290103 \| \| rs11209026 \| 0.298309 \| 0.349475 \| 0.393332 \| \| rs1128905 \| -0.56501 \| 0.742099 \| 0.44644 \| \| rs11624293 \| 1.185036 \| 0.720819 \| 0.100174 \| \| rs1250550 \| 0.391765 \| 0.772008 \| 0.61183 \| \| rs12615545 \| 0.208065 \| 0.690933 \| 0.763311 \| \| rs1801274 \| 1.011146 \| 0.691213 \| 0.143506 \| \| rs2517655 \| 0.003398 \| 0.232201 \| 0.988324 \| \| rs2531875 \| 0.175674 \| 0.666098 \| 0.791983 \| \| rs2596501 \| 0.191682 \| 0.114878 \| 0.095202 \| \| rs27529 \| -0.02579 \| 0.296605 \| 0.930706 \| \| rs2836883 \| -0.06553 \| 0.499032 \| 0.895528 \| \| rs35164067 \| 0.389343 \| 0.769033 \| 0.612663 \| \| rs4129267 \| 0.919772 \| 0.585014 \| 0.115899 \| \| rs41299637 \| 0.3969 \| 0.499325 \| 0.426688 \| \| rs4672505 \| -0.31617 \| 0.306137 \| 0.301704 \| \| rs4676410 \| 1.483912 \| 0.789996 \| 0.060329 \| \| rs6556416 \| 1.015261 \| 0.749548 \| 0.175578 \| \| rs6600247 \| 0.740105 \| 0.539088 \| 0.169789 \| \| rs7191548 \| -0.23214 \| 0.74844 \| 0.756439 \| \| rs743479 \| 0.004269 \| 0.759921 \| 0.995518 \| \| rs9901869 \| 0.626889 \| 0.548527 \| 0.253098 \| |
| --- | --- | --- | --- | --- | --- | --- | --- | --- | --- | --- | --- | --- | --- | --- | --- | --- | --- | --- | --- | --- | --- | --- | --- | --- | --- | --- | --- | --- | --- | --- | --- | --- | --- | --- | --- | --- | --- | --- | --- | --- | --- | --- | --- | --- | --- | --- | --- | --- | --- | --- | --- | --- | --- | --- | --- | --- | --- | --- | --- | --- | --- | --- | --- | --- | --- | --- | --- | --- | --- | --- | --- | --- | --- | --- | --- | --- | --- | --- | --- | --- | --- | --- | --- | --- | --- | --- | --- | --- | --- | --- | --- | --- | --- | --- | --- | --- |


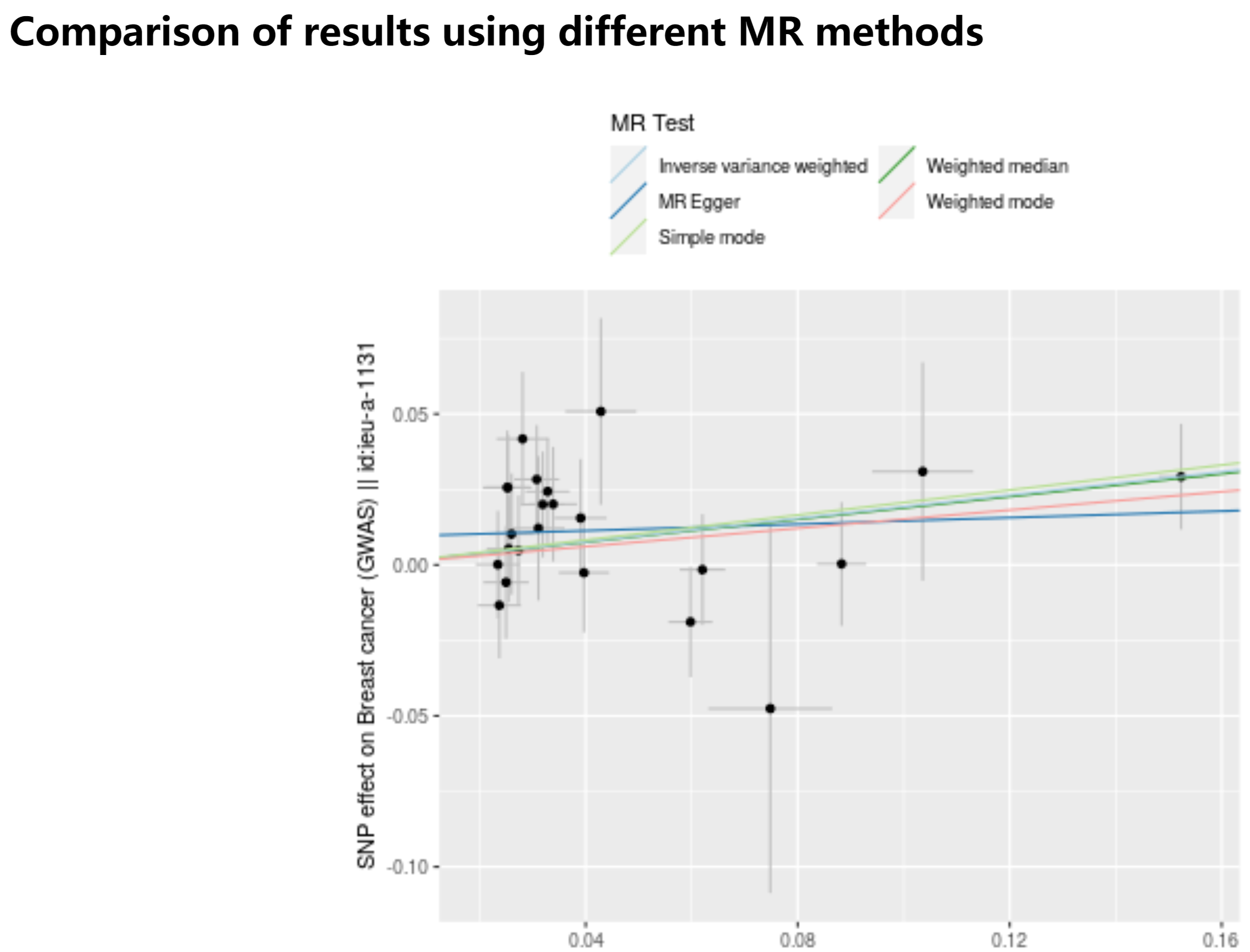


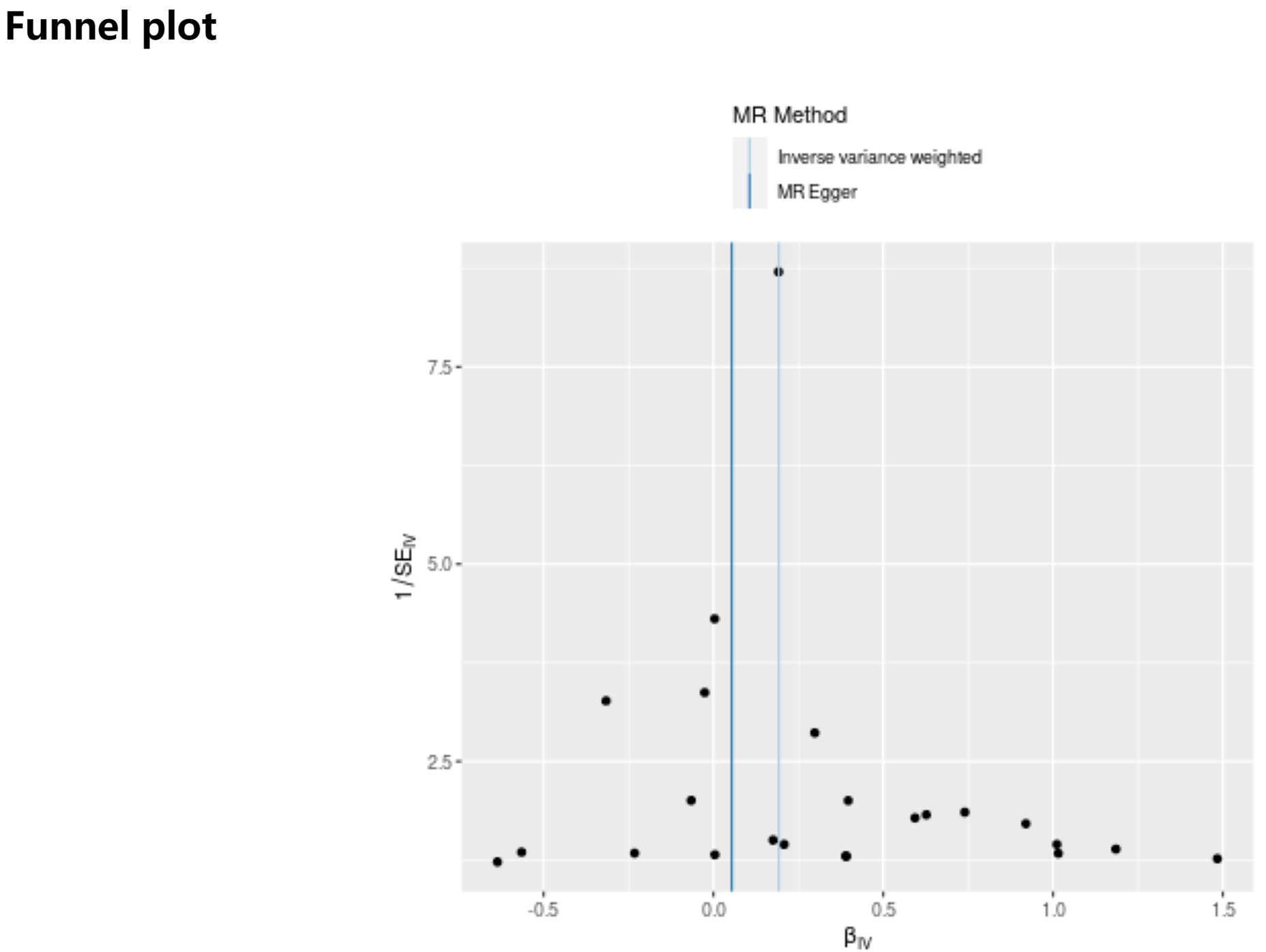

Supplement: Supplementary file 2 [file DataSheet_2.docx]
