# Supplementary material for: Diseases of the musculoskeletal system and connective tissue and risk of breast cancer: Mendelian randomization study in European and East Asian populations
Source: Front Oncol. 2023 Apr 26;13:1170119. doi: 10.3389/fonc.2023.1170119 (PMC10169740; doi:10.3389/fonc.2023.1170119)

**Two sample MR report**

**Diseases of the musculoskeletal system and connective tissue || id:finn-b-M13_MUSCULOSKELETAL against ER- Breast cancer (GWAS) || id:ieu-a-1137**

Date: **28 January, 2023**

**Results from two sample MR:**

| **method** | **nsnp** | **b** | **se** | **pval** |
| --- | --- | --- | --- | --- |
| MR Egger | 4 | 0.4565 | 0.5876 | 0.5185 |
| Weighted median | 4 | 0.4039 | 0.3475 | 0.2451 |
| Inverse variance weighted | 4 | 0.6176 | 0.2986 | 0.03863 |
| Simple mode | 4 | 0.4607 | 0.4295 | 0.3621 |
| Weighted mode | 4 | 0.4111 | 0.3621 | 0.3387 |

**Heterogeneity tests**

| **method** | **Q** | **Q_df** | **Q_pval** |
| --- | --- | --- | --- |
| MR Egger | 2.465 | 2 | 0.2916 |
| Inverse variance weighted | 2.601 | 3 | 0.4574 |

**Test for directional horizontal pleiotropy**

| **egger_intercept** | **se** | **pval** |
| --- | --- | --- |
| 0.012 | 0.036 | 0.771 |

Note - R^2^ values are approximate

Calculated as F=N-κ-1/κ × R^2^/1-R^2^

| \| SNP \| b \| se \| p \| \| --- \| --- \| --- \| --- \| \| rs113738740 \| 0.564894 \| 0.737234 \| 0.443537 \| \| rs11663824 \| 0.586735 \| 0.44898 \| 0.191274 \| \| rs148015908 \| 0.319613 \| 0.252421 \| 0.205446 \| \| rs4272793 \| -0.06107 \| 0.450382 \| 0.892143 \| |
| --- | --- | --- | --- | --- | --- | --- | --- | --- | --- | --- | --- | --- | --- | --- | --- | --- | --- | --- | --- | --- |

**Comparison of results using different MR methods**


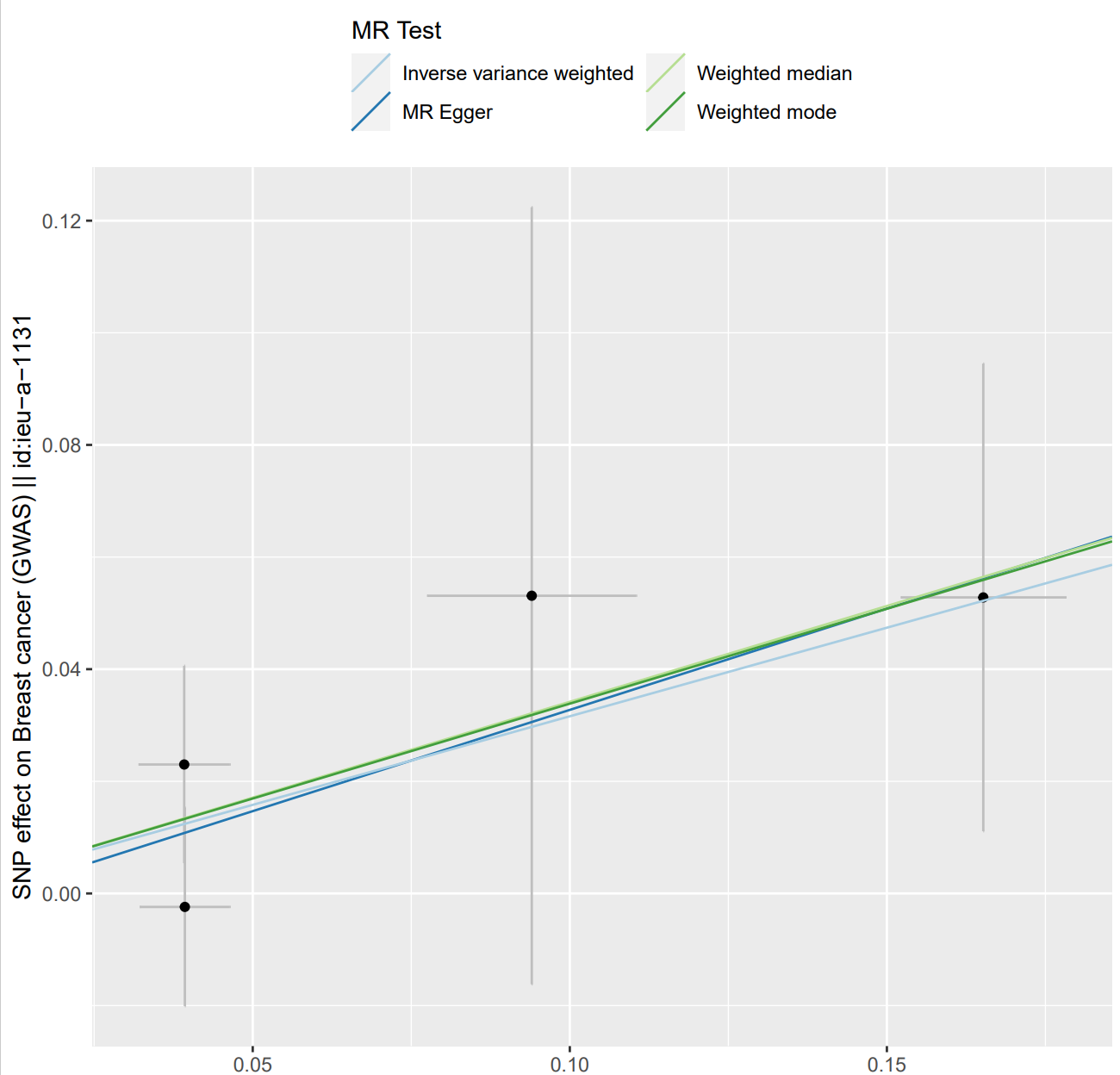


**Funnel plot**


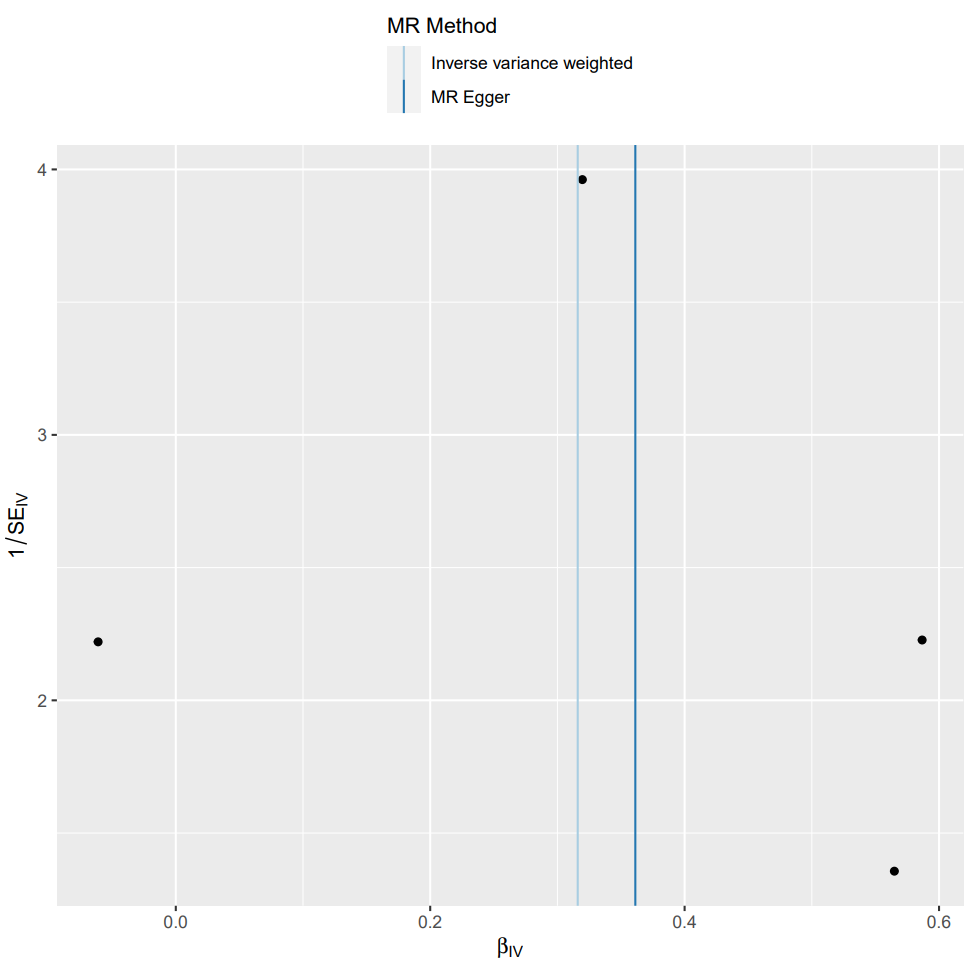

Supplement: Supplementary file 3 [file DataSheet_3.docx]
