# Supplementary material for: Diseases of the musculoskeletal system and connective tissue and risk of breast cancer: Mendelian randomization study in European and East Asian populations
Source: Front Oncol. 2023 Apr 26;13:1170119. doi: 10.3389/fonc.2023.1170119 (PMC10169740; doi:10.3389/fonc.2023.1170119)

# Two sample MR report

## Rheumatoid arthritis || id:ieu-a-831 against Breast cancer || id:bbj-a-160

Date: **29 January, 2023**

### Results from two sample MR:

| **method** | **nsnp** | **b** | **se** | **pval** |
| --- | --- | --- | --- | --- |
| MR Egger | 13 | -0.0136705 | 0.0536390 | 0.8035328 |
| Weighted median | 13 | -0.0492505 | 0.0268889 | 0.0670065 |
| Inverse variance weighted | 13 | -0.0663727 | 0.0256403 | 0.0096363 |
| Simple mode | 13 | -0.0611420 | 0.0451631 | 0.2007477 |
| Weighted mode | 13 | -0.0327942 | 0.0282857 | 0.2688469 |

### Heterogeneity tests

| **method** | **Q** | **Q_df** | **Q_pval** |
| --- | --- | --- | --- |
| MR Egger | 24.16065 | 11 | 0.0120726 |
| Inverse variance weighted | 26.89290 | 12 | 0.0080062 |

### Test for directional horizontal pleiotropy

| **egger_intercept** | **se** | **pval** |
| --- | --- | --- |
| -0.022361 | 0.0200488 | 0.2884839 |

### Test that the exposure is upstream of the outcome

| **snp_r2.exposure** | **snp_r2.outcome** | **correct_causal_direction** | **steiger_pval** |
| --- | --- | --- | --- |
| 0.0925397 | 0.0004398 | TRUE | 0 |

Note - R^2^ values are approximate

Calculated as F=N-κ-1/κ × R^2^/1-R^2^

| \| SNP \| b \| se \| p \| \| --- \| --- \| --- \| --- \| \| rs10821944 \| 0.041948 \| 0.127644 \| 0.742434 \| \| rs10946216 \| -0.11439 \| 0.081982 \| 0.162937 \| \| rs11889341 \| -0.31407 \| 0.137038 \| 0.021914 \| \| rs17427599 \| 0.021489 \| 0.032472 \| 0.508127 \| \| rs2240339 \| 0.004992 \| 0.107743 \| 0.963043 \| \| rs2244020 \| -0.13313 \| 0.062961 \| 0.034478 \| \| rs2856821 \| -0.0618 \| 0.084167 \| 0.462809 \| \| rs3734708 \| -0.03902 \| 0.113151 \| 0.730222 \| \| rs3819720 \| -0.08304 \| 0.0653 \| 0.203482 \| \| rs449635 \| -0.15268 \| 0.070761 \| 0.030957 \| \| rs58667488 \| -0.04835 \| 0.032895 \| 0.141586 \| \| rs909267 \| -0.28046 \| 0.067609 \| 3.35E-05 \| \| rs9494892 \| -0.23342 \| 0.126731 \| 0.065491 \| |
| --- | --- | --- | --- | --- | --- | --- | --- | --- | --- | --- | --- | --- | --- | --- | --- | --- | --- | --- | --- | --- | --- | --- | --- | --- | --- | --- | --- | --- | --- | --- | --- | --- | --- | --- | --- | --- | --- | --- | --- | --- | --- | --- | --- | --- | --- | --- | --- | --- | --- | --- | --- | --- | --- | --- | --- | --- |


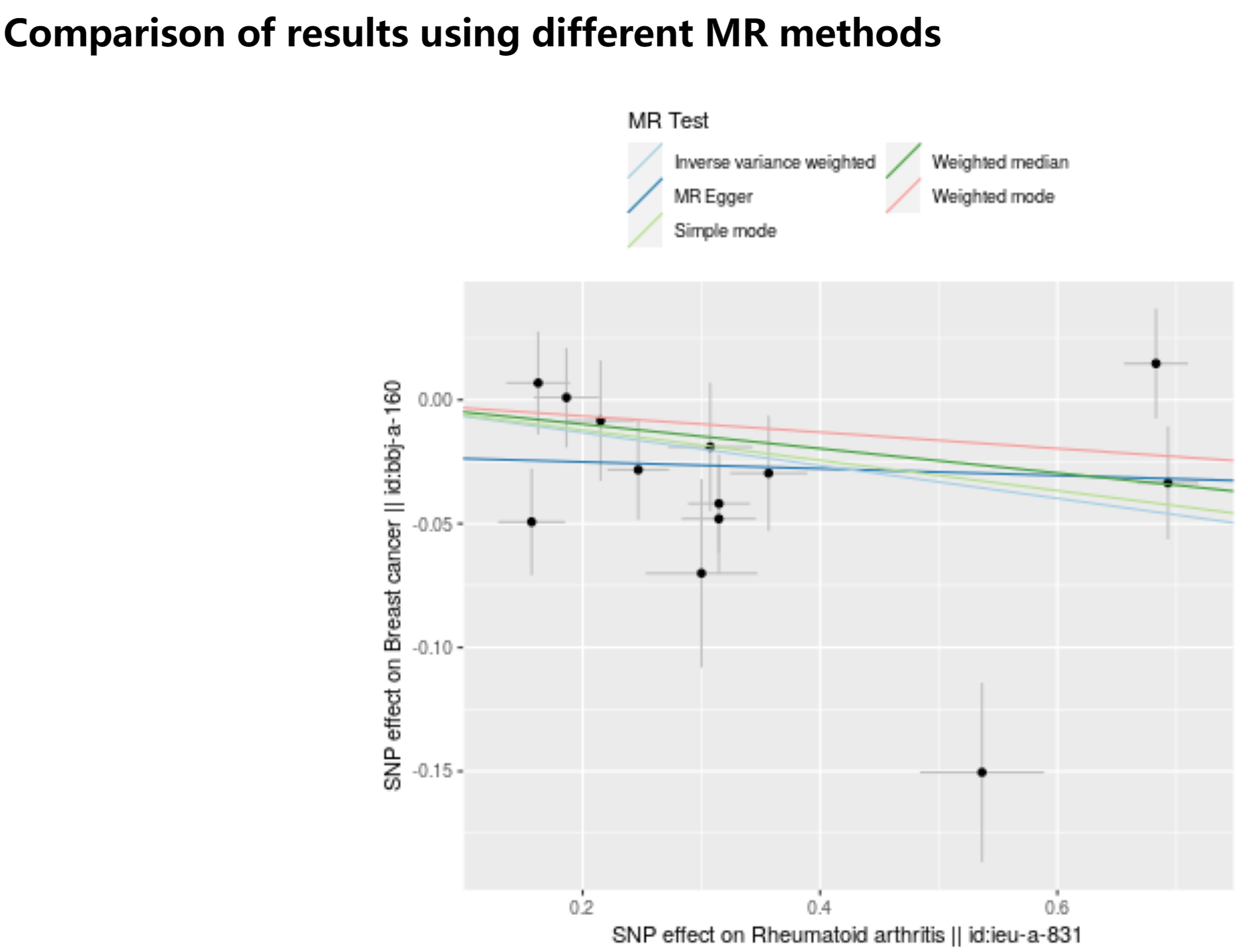

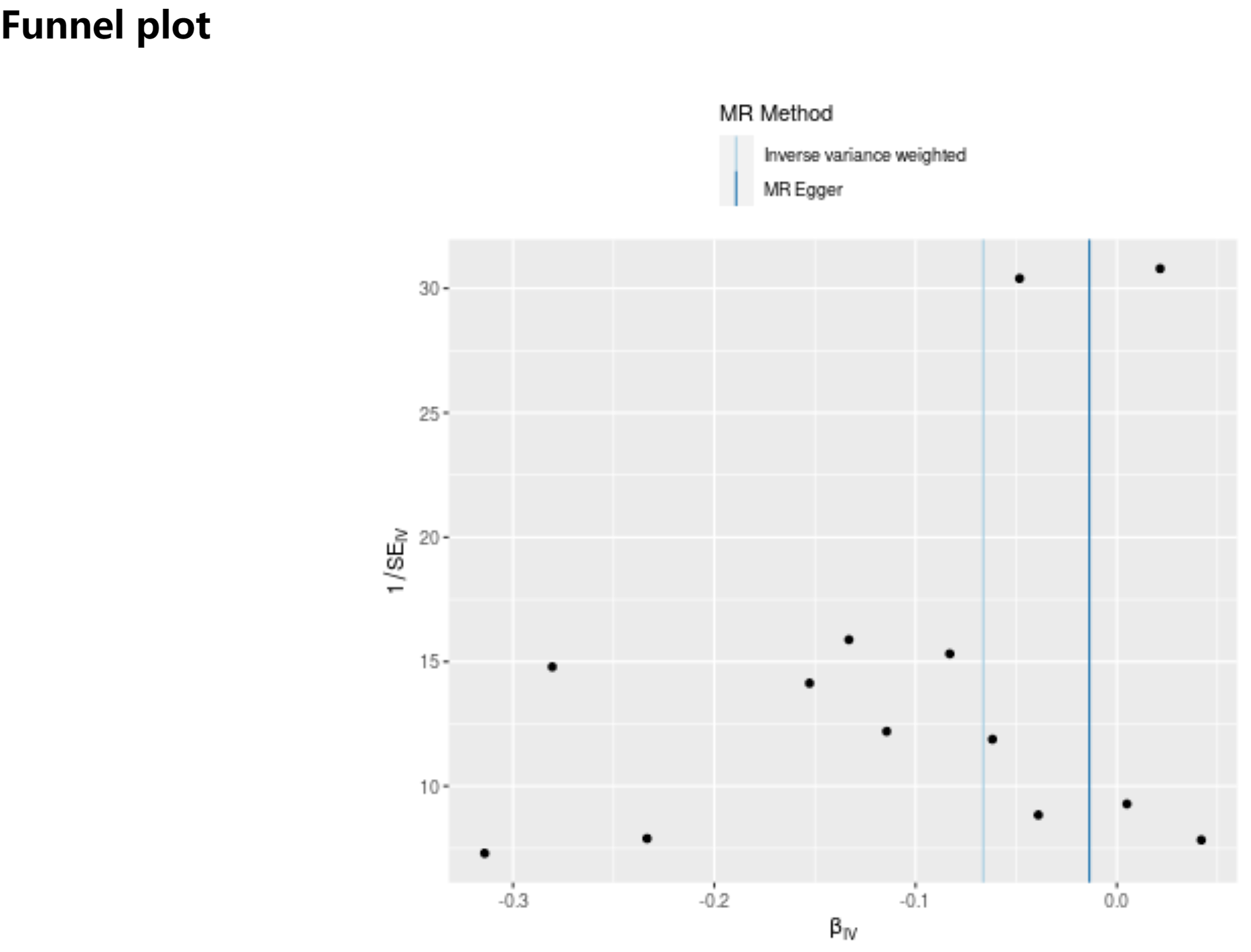

Supplement: Supplementary file 4 [file DataSheet_4.docx]
