# Supplementary material for: Diseases of the musculoskeletal system and connective tissue and risk of breast cancer: Mendelian randomization study in European and East Asian populations
Source: Front Oncol. 2023 Apr 26;13:1170119. doi: 10.3389/fonc.2023.1170119 (PMC10169740; doi:10.3389/fonc.2023.1170119)

# Two sample MR report

## Systemic lupus erythematosus || id:ebi-a-GCST90011866 against Breast cancer || id:bbj-a-160

Date: **29 January, 2023**

### Results from two sample MR:

| **method** | **nsnp** | **b** | **se** | **pval** |
| --- | --- | --- | --- | --- |
| MR Egger | 28 | -0.0660420 | 0.0532808 | 0.2262289 |
| Weighted median | 28 | -0.0764542 | 0.0247846 | 0.0020372 |
| Inverse variance weighted | 28 | -0.0502044 | 0.0182057 | 0.0058222 |
| Simple mode | 28 | -0.0351735 | 0.0450109 | 0.4413404 |
| Weighted mode | 28 | -0.0915219 | 0.0355306 | 0.0157913 |

### Heterogeneity tests

| **method** | **Q** | **Q_df** | **Q_pval** |
| --- | --- | --- | --- |
| MR Egger | 32.80185 | 26 | 0.1679248 |
| Inverse variance weighted | 32.92863 | 27 | 0.1994354 |

### Test for directional horizontal pleiotropy

| **egger_intercept** | **se** | **pval** |
| --- | --- | --- |
| 0.0046731 | 0.0147413 | 0.75377 |

### Test that the exposure is upstream of the outcome

| **snp_r2.exposure** | **snp_r2.outcome** | **correct_causal_direction** | **steiger_pval** |
| --- | --- | --- | --- |
| 0.1653143 | 0.0004801 | TRUE | 0 |

Note - R^2^ values are approximate

Calculated as F=N-κ-1/κ × R^2^/1-R^2^

| \| SNP \| b \| se \| p \| SNP \| b \| se \| p \| \| --- \| --- \| --- \| --- \| --- \| --- \| --- \| --- \| \| rs10036748 \| -0.11058 \| 0.108423 \| 0.307777 \| rs7097397 \| -0.19696 \| 0.089719 \| 0.028143 \| \| rs10516487 \| 0.037153 \| 0.127326 \| 0.770444 \| rs7486387 \| -0.2694 \| 0.121626 \| 0.026759 \| \| rs1167791 \| 0.016436 \| 0.110084 \| 0.881315 \| rs7650774 \| -0.12545 \| 0.121572 \| 0.302125 \| \| rs11889341 \| -0.11842 \| 0.05167 \| 0.021914 \| rs9387400 \| 0.22723 \| 0.118273 \| 0.054701 \| \| rs12599402 \| -0.01196 \| 0.109721 \| 0.913236 \| rs5029937 \| -0.10131 \| 0.055399 \| 0.067445 \| \| rs13213165 \| 0.008043 \| 0.07013 \| 0.908698 \| rs55701306 \| 0.029828 \| 0.129257 \| 0.817495 \| \| rs13385731 \| -0.01979 \| 0.078963 \| 0.802116 \| rs620088 \| -0.08977 \| 0.110965 \| 0.41851 \| \| rs16869875 \| -0.07936 \| 0.066911 \| 0.235612 \| rs6941485 \| 0.140422 \| 0.102926 \| 0.172473 \| \| rs16870693 \| 0.123624 \| 0.068551 \| 0.071325 \| rs6993775 \| -0.01892 \| 0.072252 \| 0.793471 \| \| rs201036579 \| -0.2593 \| 0.141098 \| 0.0661 \| rs41430444 \| -0.02709 \| 0.088013 \| 0.758212 \| \| rs244689 \| -0.02173 \| 0.144453 \| 0.8804 \| rs451263 \| -0.14629 \| 0.12275 \| 0.233353 \| \| rs2618473 \| -0.12509 \| 0.060426 \| 0.038444 \| rs4731532 \| 0.018199 \| 0.0704 \| 0.796012 \| \| rs2841281 \| -0.06735 \| 0.107688 \| 0.531665 \| rs4930642 \| -0.07831 \| 0.111151 \| 0.481087 \| \| rs3800387 \| -0.07897 \| 0.122839 \| 0.520307 \| rs4134466 \| -0.01525 \| 0.097006 \| 0.875054 \| |
| --- | --- | --- | --- | --- | --- | --- | --- | --- | --- | --- | --- | --- | --- | --- | --- | --- | --- | --- | --- | --- | --- | --- | --- | --- | --- | --- | --- | --- | --- | --- | --- | --- | --- | --- | --- | --- | --- | --- | --- | --- | --- | --- | --- | --- | --- | --- | --- | --- | --- | --- | --- | --- | --- | --- | --- | --- | --- | --- | --- | --- | --- | --- | --- | --- | --- | --- | --- | --- | --- | --- | --- | --- | --- | --- | --- | --- | --- | --- | --- | --- | --- | --- | --- | --- | --- | --- | --- | --- | --- | --- | --- | --- | --- | --- | --- | --- | --- | --- | --- | --- | --- | --- | --- | --- | --- | --- | --- | --- | --- | --- | --- | --- | --- | --- | --- | --- | --- | --- | --- | --- |


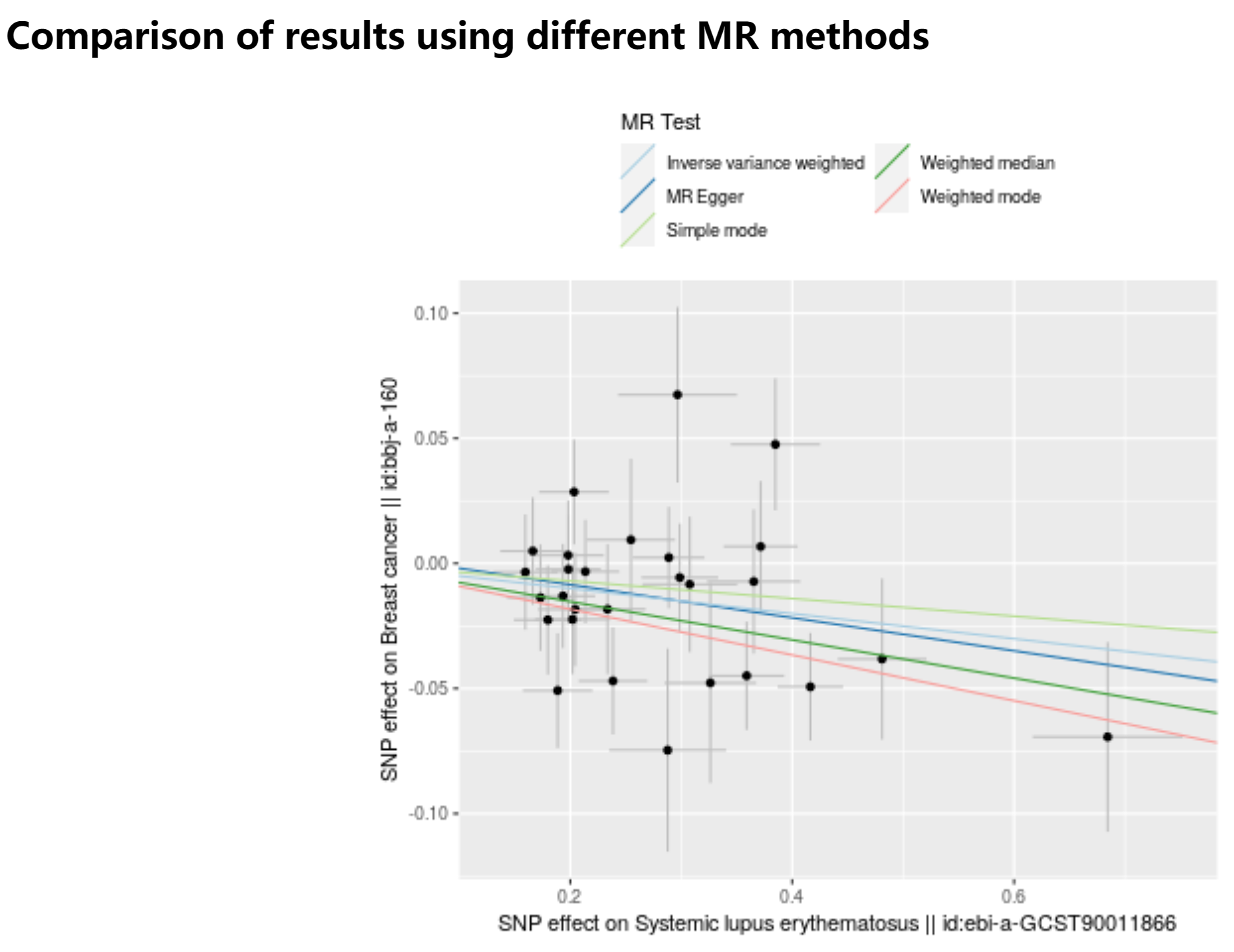

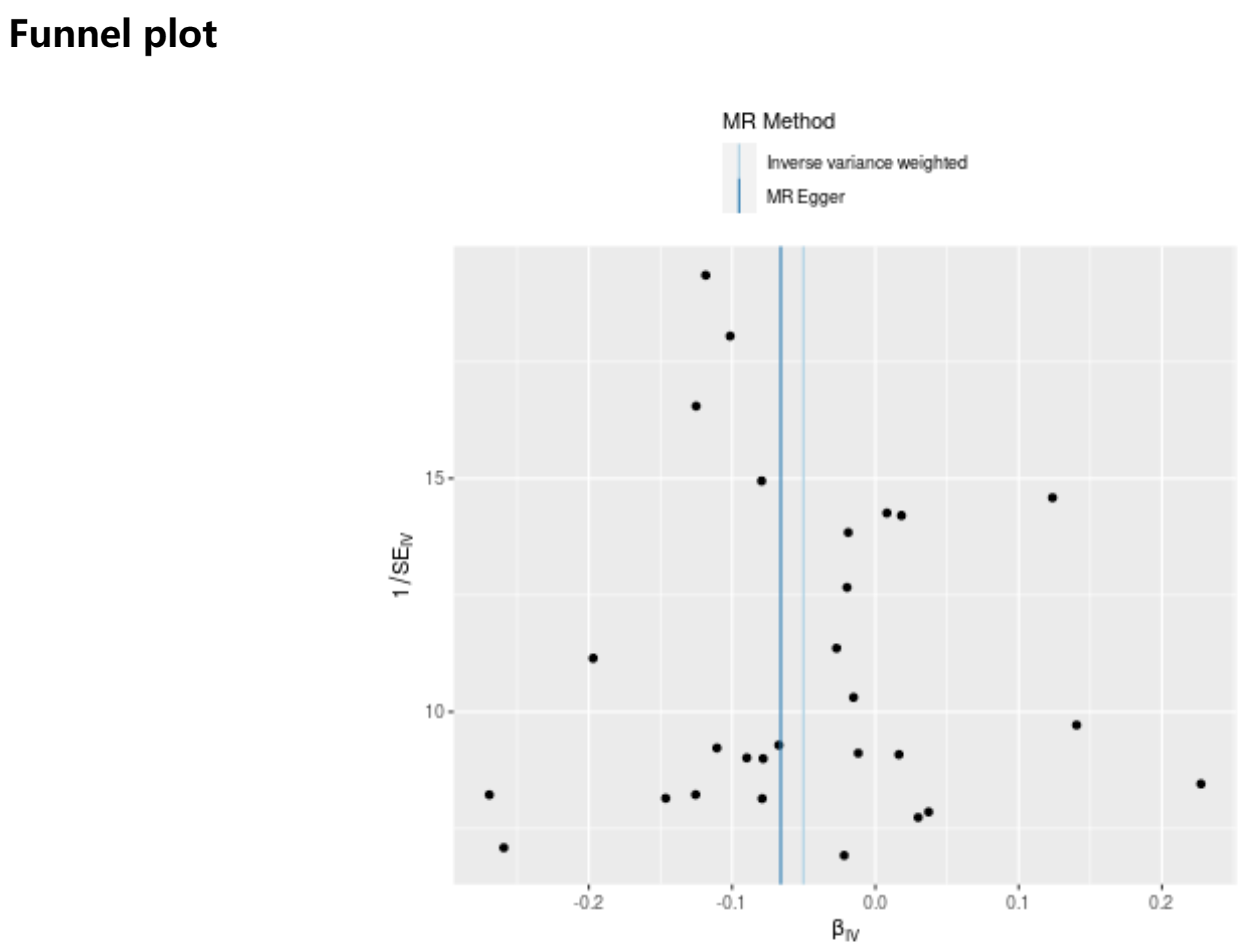

Supplement: Supplementary file 5 [file DataSheet_5.docx]
