# Supplementary material for: Diseases of the musculoskeletal system and connective tissue and risk of breast cancer: Mendelian randomization study in European and East Asian populations
Source: Front Oncol. 2023 Apr 26;13:1170119. doi: 10.3389/fonc.2023.1170119 (PMC10169740; doi:10.3389/fonc.2023.1170119)
Supplement: Supplementary file 6 [file Table_1.docx]

Supplementary Tab. 1 Selected SNP in the European population

|  | exposure | outcome | id.exposure | id.outcome | samplesize | SNP | b | se | p |
| --- | --- | --- | --- | --- | --- | --- | --- | --- | --- |
| 1 | Rheumatoid arthritis \|\| id:ebi-a-GCST90013534 | Breast cancer (GWAS) \|\| id:ieu-a-1131 | ebi-a-GCST90013534 | ieu-a-1131 | 32498 | rs10435844 | 0.40943878 | 0.23214286 | 0.07777639 |
| 2 | Rheumatoid arthritis \|\| id:ebi-a-GCST90013534 | Breast cancer (GWAS) \|\| id:ieu-a-1131 | ebi-a-GCST90013534 | ieu-a-1131 | 32498 | rs10911902 | 0.39197166 | 0.28689492 | 0.17185889 |
| 3 | Rheumatoid arthritis \|\| id:ebi-a-GCST90013534 | Breast cancer (GWAS) \|\| id:ieu-a-1131 | ebi-a-GCST90013534 | ieu-a-1131 | 32498 | rs11123811 | 0.09949749 | 0.1758794 | 0.57158802 |
| 4 | Rheumatoid arthritis \|\| id:ebi-a-GCST90013534 | Breast cancer (GWAS) \|\| id:ieu-a-1131 | ebi-a-GCST90013534 | ieu-a-1131 | 32498 | rs112733823 | 0.04136126 | 0.13560209 | 0.76035145 |
| 5 | Rheumatoid arthritis \|\| id:ebi-a-GCST90013534 | Breast cancer (GWAS) \|\| id:ieu-a-1131 | ebi-a-GCST90013534 | ieu-a-1131 | 32498 | rs114508013 | -0.2731557 | 0.11536885 | 0.01790034 |
| 6 | Rheumatoid arthritis \|\| id:ebi-a-GCST90013534 | Breast cancer (GWAS) \|\| id:ieu-a-1131 | ebi-a-GCST90013534 | ieu-a-1131 | 32498 | rs115521560 | 0.08060421 | 0.05547093 | 0.14619898 |
| 7 | Rheumatoid arthritis \|\| id:ebi-a-GCST90013534 | Breast cancer (GWAS) \|\| id:ieu-a-1131 | ebi-a-GCST90013534 | ieu-a-1131 | 32498 | rs11574914 | 0.08499566 | 0.1613183 | 0.59827573 |
| 8 | Rheumatoid arthritis \|\| id:ebi-a-GCST90013534 | Breast cancer (GWAS) \|\| id:ieu-a-1131 | ebi-a-GCST90013534 | ieu-a-1131 | 32498 | rs117026326 | -0.4506562 | 0.48950131 | 0.35723662 |
| 9 | Rheumatoid arthritis \|\| id:ebi-a-GCST90013534 | Breast cancer (GWAS) \|\| id:ieu-a-1131 | ebi-a-GCST90013534 | ieu-a-1131 | 32498 | rs11754264 | -0.0206034 | 0.3473142 | 0.95269561 |
| 10 | Rheumatoid arthritis \|\| id:ebi-a-GCST90013534 | Breast cancer (GWAS) \|\| id:ieu-a-1131 | ebi-a-GCST90013534 | ieu-a-1131 | 32498 | rs11889341 | 0.07844475 | 0.14392906 | 0.58573722 |
| 11 | Rheumatoid arthritis \|\| id:ebi-a-GCST90013534 | Breast cancer (GWAS) \|\| id:ieu-a-1131 | ebi-a-GCST90013534 | ieu-a-1131 | 32498 | rs12126142 | 0.37683089 | 0.23968043 | 0.11589902 |
| 12 | Rheumatoid arthritis \|\| id:ebi-a-GCST90013534 | Breast cancer (GWAS) \|\| id:ieu-a-1131 | ebi-a-GCST90013534 | ieu-a-1131 | 32498 | rs1234313 | -0.0627353 | 0.23462986 | 0.78917684 |
| 13 | Rheumatoid arthritis \|\| id:ebi-a-GCST90013534 | Breast cancer (GWAS) \|\| id:ieu-a-1131 | ebi-a-GCST90013534 | ieu-a-1131 | 32498 | rs12466919 | -0.2507317 | 0.17658537 | 0.15563985 |
| 14 | Rheumatoid arthritis \|\| id:ebi-a-GCST90013534 | Breast cancer (GWAS) \|\| id:ieu-a-1131 | ebi-a-GCST90013534 | ieu-a-1131 | 32498 | rs12530098 | 0.44283647 | 0.28437048 | 0.11941069 |
| 15 | Rheumatoid arthritis \|\| id:ebi-a-GCST90013534 | Breast cancer (GWAS) \|\| id:ieu-a-1131 | ebi-a-GCST90013534 | ieu-a-1131 | 32498 | rs12918327 | 0.17416378 | 0.29065744 | 0.54903529 |
| 16 | Rheumatoid arthritis \|\| id:ebi-a-GCST90013534 | Breast cancer (GWAS) \|\| id:ieu-a-1131 | ebi-a-GCST90013534 | ieu-a-1131 | 32498 | rs13103285 | 0.25075834 | 0.17694641 | 0.15644116 |
| 17 | Rheumatoid arthritis \|\| id:ebi-a-GCST90013534 | Breast cancer (GWAS) \|\| id:ieu-a-1131 | ebi-a-GCST90013534 | ieu-a-1131 | 32498 | rs1355208 | 0.55867971 | 0.22249389 | 0.01203934 |
| 18 | Rheumatoid arthritis \|\| id:ebi-a-GCST90013534 | Breast cancer (GWAS) \|\| id:ieu-a-1131 | ebi-a-GCST90013534 | ieu-a-1131 | 32498 | rs139395255 | -0.1220976 | 0.17401513 | 0.48289798 |
| 19 | Rheumatoid arthritis \|\| id:ebi-a-GCST90013534 | Breast cancer (GWAS) \|\| id:ieu-a-1131 | ebi-a-GCST90013534 | ieu-a-1131 | 32498 | rs146305655 | 0.19616351 | 0.10710208 | 0.06701752 |
| 20 | Rheumatoid arthritis \|\| id:ebi-a-GCST90013534 | Breast cancer (GWAS) \|\| id:ieu-a-1131 | ebi-a-GCST90013534 | ieu-a-1131 | 32498 | rs1538981 | -0.438152 | 0.26080477 | 0.09295732 |
| 21 | Rheumatoid arthritis \|\| id:ebi-a-GCST90013534 | Breast cancer (GWAS) \|\| id:ieu-a-1131 | ebi-a-GCST90013534 | ieu-a-1131 | 32498 | rs1571878 | -0.0337882 | 0.11500975 | 0.76892196 |
| 22 | Rheumatoid arthritis \|\| id:ebi-a-GCST90013534 | Breast cancer (GWAS) \|\| id:ieu-a-1131 | ebi-a-GCST90013534 | ieu-a-1131 | 32498 | rs1595260 | -0.2686391 | 0.23076923 | 0.24438245 |
| 23 | Rheumatoid arthritis \|\| id:ebi-a-GCST90013534 | Breast cancer (GWAS) \|\| id:ieu-a-1131 | ebi-a-GCST90013534 | ieu-a-1131 | 32498 | rs1611236 | 0.15708155 | 0.16051502 | 0.3277729 |
| 24 | Rheumatoid arthritis \|\| id:ebi-a-GCST90013534 | Breast cancer (GWAS) \|\| id:ieu-a-1131 | ebi-a-GCST90013534 | ieu-a-1131 | 32498 | rs1858037 | -0.0395257 | 0.18181818 | 0.82790341 |
| 25 | Rheumatoid arthritis \|\| id:ebi-a-GCST90013534 | Breast cancer (GWAS) \|\| id:ieu-a-1131 | ebi-a-GCST90013534 | ieu-a-1131 | 32498 | rs1883832 | -0.21673 | 0.19201521 | 0.25901898 |
| 26 | Rheumatoid arthritis \|\| id:ebi-a-GCST90013534 | Breast cancer (GWAS) \|\| id:ieu-a-1131 | ebi-a-GCST90013534 | ieu-a-1131 | 32498 | rs1893592 | 0.15778689 | 0.19672131 | 0.42250476 |
| 27 | Rheumatoid arthritis \|\| id:ebi-a-GCST90013534 | Breast cancer (GWAS) \|\| id:ieu-a-1131 | ebi-a-GCST90013534 | ieu-a-1131 | 32498 | rs1950897 | -0.2974743 | 0.17867166 | 0.09592842 |
| 28 | Rheumatoid arthritis \|\| id:ebi-a-GCST90013534 | Breast cancer (GWAS) \|\| id:ieu-a-1131 | ebi-a-GCST90013534 | ieu-a-1131 | 32498 | rs2069235 | 0.00462963 | 0.15046296 | 0.97545358 |
| 29 | Rheumatoid arthritis \|\| id:ebi-a-GCST90013534 | Breast cancer (GWAS) \|\| id:ieu-a-1131 | ebi-a-GCST90013534 | ieu-a-1131 | 32498 | rs2073609 | -0.2701652 | 0.58114674 | 0.64201529 |
| 30 | Rheumatoid arthritis \|\| id:ebi-a-GCST90013534 | Breast cancer (GWAS) \|\| id:ieu-a-1131 | ebi-a-GCST90013534 | ieu-a-1131 | 32498 | rs2076616 | 0.12655367 | 0.21355932 | 0.55345384 |
| 31 | Rheumatoid arthritis \|\| id:ebi-a-GCST90013534 | Breast cancer (GWAS) \|\| id:ieu-a-1131 | ebi-a-GCST90013534 | ieu-a-1131 | 32498 | rs212389 | -0.0746692 | 0.1710775 | 0.66250005 |
| 32 | Rheumatoid arthritis \|\| id:ebi-a-GCST90013534 | Breast cancer (GWAS) \|\| id:ieu-a-1131 | ebi-a-GCST90013534 | ieu-a-1131 | 32498 | rs2233424 | 0.11405295 | 0.2311609 | 0.62173558 |
| 33 | Rheumatoid arthritis \|\| id:ebi-a-GCST90013534 | Breast cancer (GWAS) \|\| id:ieu-a-1131 | ebi-a-GCST90013534 | ieu-a-1131 | 32498 | rs2258734 | 0.3072747 | 0.2062975 | 0.13636268 |
| 34 | Rheumatoid arthritis \|\| id:ebi-a-GCST90013534 | Breast cancer (GWAS) \|\| id:ieu-a-1131 | ebi-a-GCST90013534 | ieu-a-1131 | 32498 | rs2275806 | 0.15310345 | 0.24689655 | 0.53518423 |
| 35 | Rheumatoid arthritis \|\| id:ebi-a-GCST90013534 | Breast cancer (GWAS) \|\| id:ieu-a-1131 | ebi-a-GCST90013534 | ieu-a-1131 | 32498 | rs2301888 | 0.33307332 | 0.14352574 | 0.02030562 |
| 36 | Rheumatoid arthritis \|\| id:ebi-a-GCST90013534 | Breast cancer (GWAS) \|\| id:ieu-a-1131 | ebi-a-GCST90013534 | ieu-a-1131 | 32498 | rs244685 | -0.0247191 | 0.28426966 | 0.93070607 |
| 37 | Rheumatoid arthritis \|\| id:ebi-a-GCST90013534 | Breast cancer (GWAS) \|\| id:ieu-a-1131 | ebi-a-GCST90013534 | ieu-a-1131 | 32498 | rs28411352 | 0.18818381 | 0.22647702 | 0.40602003 |
| 38 | Rheumatoid arthritis \|\| id:ebi-a-GCST90013534 | Breast cancer (GWAS) \|\| id:ieu-a-1131 | ebi-a-GCST90013534 | ieu-a-1131 | 32498 | rs2841275 | -0.126778 | 0.12121212 | 0.29559872 |
| 39 | Rheumatoid arthritis \|\| id:ebi-a-GCST90013534 | Breast cancer (GWAS) \|\| id:ieu-a-1131 | ebi-a-GCST90013534 | ieu-a-1131 | 32498 | rs28421442 | 0.0623987 | 0.58427877 | 0.91495073 |
| 40 | Rheumatoid arthritis \|\| id:ebi-a-GCST90013534 | Breast cancer (GWAS) \|\| id:ieu-a-1131 | ebi-a-GCST90013534 | ieu-a-1131 | 32498 | rs2847297 | 0.02990033 | 0.20487265 | 0.88396406 |
| 41 | Rheumatoid arthritis \|\| id:ebi-a-GCST90013534 | Breast cancer (GWAS) \|\| id:ieu-a-1131 | ebi-a-GCST90013534 | ieu-a-1131 | 32498 | rs2918392 | 0.14670659 | 0.27245509 | 0.59025845 |
| 42 | Rheumatoid arthritis \|\| id:ebi-a-GCST90013534 | Breast cancer (GWAS) \|\| id:ieu-a-1131 | ebi-a-GCST90013534 | ieu-a-1131 | 32498 | rs3025669 | -0.3480663 | 0.4629045 | 0.45210028 |
| 43 | Rheumatoid arthritis \|\| id:ebi-a-GCST90013534 | Breast cancer (GWAS) \|\| id:ieu-a-1131 | ebi-a-GCST90013534 | ieu-a-1131 | 32498 | rs3087243 | -0.2093577 | 0.14036479 | 0.1358236 |
| 44 | Rheumatoid arthritis \|\| id:ebi-a-GCST90013534 | Breast cancer (GWAS) \|\| id:ieu-a-1131 | ebi-a-GCST90013534 | ieu-a-1131 | 32498 | rs3134883 | -0.1523713 | 0.18970737 | 0.42186406 |
| 45 | Rheumatoid arthritis \|\| id:ebi-a-GCST90013534 | Breast cancer (GWAS) \|\| id:ieu-a-1131 | ebi-a-GCST90013534 | ieu-a-1131 | 32498 | rs34046593 | 0.04571027 | 0.13361463 | 0.73227168 |
| 46 | Rheumatoid arthritis \|\| id:ebi-a-GCST90013534 | Breast cancer (GWAS) \|\| id:ieu-a-1131 | ebi-a-GCST90013534 | ieu-a-1131 | 32498 | rs34502849 | 0.00822562 | 0.21151586 | 0.96897898 |
| 47 | Rheumatoid arthritis \|\| id:ebi-a-GCST90013534 | Breast cancer (GWAS) \|\| id:ieu-a-1131 | ebi-a-GCST90013534 | ieu-a-1131 | 32498 | rs3757387 | -0.1302589 | 0.15695793 | 0.40659707 |
| 48 | Rheumatoid arthritis \|\| id:ebi-a-GCST90013534 | Breast cancer (GWAS) \|\| id:ieu-a-1131 | ebi-a-GCST90013534 | ieu-a-1131 | 32498 | rs3761959 | -0.0201613 | 0.23655914 | 0.93208071 |
| 49 | Rheumatoid arthritis \|\| id:ebi-a-GCST90013534 | Breast cancer (GWAS) \|\| id:ieu-a-1131 | ebi-a-GCST90013534 | ieu-a-1131 | 32498 | rs3806624 | 0.09501738 | 0.20393975 | 0.64128057 |
| 50 | Rheumatoid arthritis \|\| id:ebi-a-GCST90013534 | Breast cancer (GWAS) \|\| id:ieu-a-1131 | ebi-a-GCST90013534 | ieu-a-1131 | 32498 | rs403214 | 0.35886214 | 0.20678337 | 0.08266118 |
| 51 | Rheumatoid arthritis \|\| id:ebi-a-GCST90013534 | Breast cancer (GWAS) \|\| id:ieu-a-1131 | ebi-a-GCST90013534 | ieu-a-1131 | 32498 | rs42034 | 0.5097589 | 0.23191734 | 0.02794769 |
| 52 | Rheumatoid arthritis \|\| id:ebi-a-GCST90013534 | Breast cancer (GWAS) \|\| id:ieu-a-1131 | ebi-a-GCST90013534 | ieu-a-1131 | 32498 | rs4409785 | -0.1507128 | 0.23828921 | 0.52707417 |
| 53 | Rheumatoid arthritis \|\| id:ebi-a-GCST90013534 | Breast cancer (GWAS) \|\| id:ieu-a-1131 | ebi-a-GCST90013534 | ieu-a-1131 | 32498 | rs4602367 | 0.04533333 | 0.232 | 0.84507802 |
| 54 | Rheumatoid arthritis \|\| id:ebi-a-GCST90013534 | Breast cancer (GWAS) \|\| id:ieu-a-1131 | ebi-a-GCST90013534 | ieu-a-1131 | 32498 | rs4717901 | -0.4208835 | 0.38714859 | 0.2769763 |
| 55 | Rheumatoid arthritis \|\| id:ebi-a-GCST90013534 | Breast cancer (GWAS) \|\| id:ieu-a-1131 | ebi-a-GCST90013534 | ieu-a-1131 | 32498 | rs4795400 | 0.08479139 | 0.23687752 | 0.72037733 |
| 56 | Rheumatoid arthritis \|\| id:ebi-a-GCST90013534 | Breast cancer (GWAS) \|\| id:ieu-a-1131 | ebi-a-GCST90013534 | ieu-a-1131 | 32498 | rs5020946 | 0.03037276 | 0.02929897 | 0.29989937 |
| 57 | Rheumatoid arthritis \|\| id:ebi-a-GCST90013534 | Breast cancer (GWAS) \|\| id:ieu-a-1131 | ebi-a-GCST90013534 | ieu-a-1131 | 32498 | rs502919 | -0.4004825 | 0.26899879 | 0.13654288 |
| 58 | Rheumatoid arthritis \|\| id:ebi-a-GCST90013534 | Breast cancer (GWAS) \|\| id:ieu-a-1131 | ebi-a-GCST90013534 | ieu-a-1131 | 32498 | rs5754104 | 0.27609428 | 0.25028058 | 0.2699668 |
| 59 | Rheumatoid arthritis \|\| id:ebi-a-GCST90013534 | Breast cancer (GWAS) \|\| id:ieu-a-1131 | ebi-a-GCST90013534 | ieu-a-1131 | 32498 | rs6011186 | 0.26815642 | 0.36126629 | 0.45792495 |
| 60 | Rheumatoid arthritis \|\| id:ebi-a-GCST90013534 | Breast cancer (GWAS) \|\| id:ieu-a-1131 | ebi-a-GCST90013534 | ieu-a-1131 | 32498 | rs61828284 | 0.24231913 | 0.19573835 | 0.21572545 |
| 61 | Rheumatoid arthritis \|\| id:ebi-a-GCST90013534 | Breast cancer (GWAS) \|\| id:ieu-a-1131 | ebi-a-GCST90013534 | ieu-a-1131 | 32498 | rs62422878 | 0.024108 | 0.21504339 | 0.91073807 |
| 62 | Rheumatoid arthritis \|\| id:ebi-a-GCST90013534 | Breast cancer (GWAS) \|\| id:ieu-a-1131 | ebi-a-GCST90013534 | ieu-a-1131 | 32498 | rs6421571 | -0.1783582 | 0.17835821 | 0.31731051 |
| 63 | Rheumatoid arthritis \|\| id:ebi-a-GCST90013534 | Breast cancer (GWAS) \|\| id:ieu-a-1131 | ebi-a-GCST90013534 | ieu-a-1131 | 32498 | rs6479800 | -0.1381032 | 0.17470882 | 0.42924972 |
| 64 | Rheumatoid arthritis \|\| id:ebi-a-GCST90013534 | Breast cancer (GWAS) \|\| id:ieu-a-1131 | ebi-a-GCST90013534 | ieu-a-1131 | 32498 | rs660442 | 0.39643861 | 0.20243674 | 0.05019091 |
| 65 | Rheumatoid arthritis \|\| id:ebi-a-GCST90013534 | Breast cancer (GWAS) \|\| id:ieu-a-1131 | ebi-a-GCST90013534 | ieu-a-1131 | 32498 | rs6679677 | 0.14686971 | 0.05228426 | 0.00496862 |
| 66 | Rheumatoid arthritis \|\| id:ebi-a-GCST90013534 | Breast cancer (GWAS) \|\| id:ieu-a-1131 | ebi-a-GCST90013534 | ieu-a-1131 | 32498 | rs7097397 | -0.3565525 | 0.21723731 | 0.10073425 |
| 67 | Rheumatoid arthritis \|\| id:ebi-a-GCST90013534 | Breast cancer (GWAS) \|\| id:ieu-a-1131 | ebi-a-GCST90013534 | ieu-a-1131 | 32498 | rs7105899 | -0.3111669 | 0.22459222 | 0.16590731 |
| 68 | Rheumatoid arthritis \|\| id:ebi-a-GCST90013534 | Breast cancer (GWAS) \|\| id:ieu-a-1131 | ebi-a-GCST90013534 | ieu-a-1131 | 32498 | rs71508903 | -0.0390047 | 0.15063887 | 0.79569035 |
| 69 | Rheumatoid arthritis \|\| id:ebi-a-GCST90013534 | Breast cancer (GWAS) \|\| id:ieu-a-1131 | ebi-a-GCST90013534 | ieu-a-1131 | 32498 | rs71565312 | 0.35479256 | 0.13462089 | 0.00840149 |
| 70 | Rheumatoid arthritis \|\| id:ebi-a-GCST90013534 | Breast cancer (GWAS) \|\| id:ieu-a-1131 | ebi-a-GCST90013534 | ieu-a-1131 | 32498 | rs7170107 | 0.01683748 | 0.14494876 | 0.90752446 |
| 71 | Rheumatoid arthritis \|\| id:ebi-a-GCST90013534 | Breast cancer (GWAS) \|\| id:ieu-a-1131 | ebi-a-GCST90013534 | ieu-a-1131 | 32498 | rs7206670 | 0.28673324 | 0.26105563 | 0.27204704 |
| 72 | Rheumatoid arthritis \|\| id:ebi-a-GCST90013534 | Breast cancer (GWAS) \|\| id:ieu-a-1131 | ebi-a-GCST90013534 | ieu-a-1131 | 32498 | rs740122 | 0.78772379 | 0.2685422 | 0.00335344 |
| 73 | Rheumatoid arthritis \|\| id:ebi-a-GCST90013534 | Breast cancer (GWAS) \|\| id:ieu-a-1131 | ebi-a-GCST90013534 | ieu-a-1131 | 32498 | rs76153210 | 0.12273012 | 0.39261115 | 0.75458482 |
| 74 | Rheumatoid arthritis \|\| id:ebi-a-GCST90013534 | Breast cancer (GWAS) \|\| id:ieu-a-1131 | ebi-a-GCST90013534 | ieu-a-1131 | 32498 | rs7731626 | 0.02607362 | 0.099182 | 0.79263799 |
| 75 | Rheumatoid arthritis \|\| id:ebi-a-GCST90013534 | Breast cancer (GWAS) \|\| id:ieu-a-1131 | ebi-a-GCST90013534 | ieu-a-1131 | 32498 | rs7749323 | 0.00458554 | 0.19223986 | 0.9809697 |
| 76 | Rheumatoid arthritis \|\| id:ebi-a-GCST90013534 | Breast cancer (GWAS) \|\| id:ieu-a-1131 | ebi-a-GCST90013534 | ieu-a-1131 | 32498 | rs8032939 | -0.0184887 | 0.16318328 | 0.90979233 |
| 77 | Rheumatoid arthritis \|\| id:ebi-a-GCST90013534 | Breast cancer (GWAS) \|\| id:ieu-a-1131 | ebi-a-GCST90013534 | ieu-a-1131 | 32498 | rs8126756 | -0.4665857 | 0.31105711 | 0.1336144 |
| 78 | Rheumatoid arthritis \|\| id:ebi-a-GCST90013534 | Breast cancer (GWAS) \|\| id:ieu-a-1131 | ebi-a-GCST90013534 | ieu-a-1131 | 32498 | rs9271365 | 0.01779869 | 0.0413257 | 0.66669156 |
| 79 | Rheumatoid arthritis \|\| id:ebi-a-GCST90013534 | Breast cancer (GWAS) \|\| id:ieu-a-1131 | ebi-a-GCST90013534 | ieu-a-1131 | 32498 | rs9405192 | 0.32808989 | 0.27977528 | 0.24091984 |
| 80 | Rheumatoid arthritis \|\| id:ebi-a-GCST90013534 | Breast cancer (GWAS) \|\| id:ieu-a-1131 | ebi-a-GCST90013534 | ieu-a-1131 | 32498 | rs9532434 | -0.0342105 | 0.16052632 | 0.83123746 |
| 81 | Rheumatoid arthritis \|\| id:ebi-a-GCST90013534 | Breast cancer (GWAS) \|\| id:ieu-a-1131 | ebi-a-GCST90013534 | ieu-a-1131 | 32498 | rs9693589 | -0.148181 | 0.18012422 | 0.41070131 |
| 82 | Rheumatoid arthritis \|\| id:ebi-a-GCST90013534 | Breast cancer (GWAS) \|\| id:ieu-a-1131 | ebi-a-GCST90013534 | ieu-a-1131 | 32498 | rs9927316 | -0.0309051 | 0.25055188 | 0.90183152 |
| 83 | Rheumatoid arthritis \|\| id:ebi-a-GCST90013534 | Breast cancer (GWAS) \|\| id:ieu-a-1131 | ebi-a-GCST90013534 | ieu-a-1131 | 32498 | rs9943599 | -0.4337349 | 0.21445783 | 0.04312762 |
| 84 | Rheumatoid arthritis \|\| id:ebi-a-GCST90013534 | Breast cancer (GWAS) \|\| id:ieu-a-1131 | ebi-a-GCST90013534 | ieu-a-1131 | 32498 | All - Inverse variance weighted | 0.03909264 | 0.01715935 | 0.02271397 |
| 85 | Rheumatoid arthritis \|\| id:ebi-a-GCST90013534 | Breast cancer (GWAS) \|\| id:ieu-a-1131 | ebi-a-GCST90013534 | ieu-a-1131 | 32498 | All - MR Egger | 0.0467231 | 0.02688457 | 0.08602617 |
| 1 | Systemic lupus erythematosus \|\| id:ebi-a-GCST003156 | Breast cancer (GWAS) \|\| id:ieu-a-1131 | ebi-a-GCST003156 | ieu-a-1131 | 32498 | rs10048743 | -0.0220672 | 0.10903804 | 0.83961892 |
| 2 | Systemic lupus erythematosus \|\| id:ebi-a-GCST003156 | Breast cancer (GWAS) \|\| id:ieu-a-1131 | ebi-a-GCST003156 | ieu-a-1131 | 32498 | rs10200680 | -0.188762 | 0.10746153 | 0.07899381 |
| 3 | Systemic lupus erythematosus \|\| id:ebi-a-GCST003156 | Breast cancer (GWAS) \|\| id:ieu-a-1131 | ebi-a-GCST003156 | ieu-a-1131 | 32498 | rs1078324 | 0.04121399 | 0.05411089 | 0.44626413 |
| 4 | Systemic lupus erythematosus \|\| id:ebi-a-GCST003156 | Breast cancer (GWAS) \|\| id:ieu-a-1131 | ebi-a-GCST003156 | ieu-a-1131 | 32498 | rs10912578 | -0.0153933 | 0.07615653 | 0.83981692 |
| 5 | Systemic lupus erythematosus \|\| id:ebi-a-GCST003156 | Breast cancer (GWAS) \|\| id:ieu-a-1131 | ebi-a-GCST003156 | ieu-a-1131 | 32498 | rs1143679 | -0.0291988 | 0.06148921 | 0.63488652 |
| 6 | Systemic lupus erythematosus \|\| id:ebi-a-GCST003156 | Breast cancer (GWAS) \|\| id:ieu-a-1131 | ebi-a-GCST003156 | ieu-a-1131 | 32498 | rs12094036 | 0.0502277 | 0.10258627 | 0.6244069 |
| 7 | Systemic lupus erythematosus \|\| id:ebi-a-GCST003156 | Breast cancer (GWAS) \|\| id:ieu-a-1131 | ebi-a-GCST003156 | ieu-a-1131 | 32498 | rs12524498 | 0.20123414 | 0.10425562 | 0.05358213 |
| 8 | Systemic lupus erythematosus \|\| id:ebi-a-GCST003156 | Breast cancer (GWAS) \|\| id:ieu-a-1131 | ebi-a-GCST003156 | ieu-a-1131 | 32498 | rs13019891 | -0.0144098 | 0.03148799 | 0.64722036 |
| 9 | Systemic lupus erythematosus \|\| id:ebi-a-GCST003156 | Breast cancer (GWAS) \|\| id:ieu-a-1131 | ebi-a-GCST003156 | ieu-a-1131 | 32498 | rs13136219 | -0.069973 | 0.10381238 | 0.50029024 |
| 10 | Systemic lupus erythematosus \|\| id:ebi-a-GCST003156 | Breast cancer (GWAS) \|\| id:ieu-a-1131 | ebi-a-GCST003156 | ieu-a-1131 | 32498 | rs13332649 | 0.11947469 | 0.0686344 | 0.08172903 |
| 11 | Systemic lupus erythematosus \|\| id:ebi-a-GCST003156 | Breast cancer (GWAS) \|\| id:ieu-a-1131 | ebi-a-GCST003156 | ieu-a-1131 | 32498 | rs143123127 | -0.1682964 | 0.10127573 | 0.09655996 |
| 12 | Systemic lupus erythematosus \|\| id:ebi-a-GCST003156 | Breast cancer (GWAS) \|\| id:ieu-a-1131 | ebi-a-GCST003156 | ieu-a-1131 | 32498 | rs1464446 | -0.0213087 | 0.06879673 | 0.75676285 |
| 13 | Systemic lupus erythematosus \|\| id:ebi-a-GCST003156 | Breast cancer (GWAS) \|\| id:ieu-a-1131 | ebi-a-GCST003156 | ieu-a-1131 | 32498 | rs150180633 | 0.06173112 | 0.10116147 | 0.54171367 |
| 14 | Systemic lupus erythematosus \|\| id:ebi-a-GCST003156 | Breast cancer (GWAS) \|\| id:ieu-a-1131 | ebi-a-GCST003156 | ieu-a-1131 | 32498 | rs17849501 | -0.0110984 | 0.06535706 | 0.86515852 |
| 15 | Systemic lupus erythematosus \|\| id:ebi-a-GCST003156 | Breast cancer (GWAS) \|\| id:ieu-a-1131 | ebi-a-GCST003156 | ieu-a-1131 | 32498 | rs2431697 | -0.1339942 | 0.07842469 | 0.08753036 |
| 16 | Systemic lupus erythematosus \|\| id:ebi-a-GCST003156 | Breast cancer (GWAS) \|\| id:ieu-a-1131 | ebi-a-GCST003156 | ieu-a-1131 | 32498 | rs2459611 | -0.1205211 | 0.11019073 | 0.27406464 |
| 17 | Systemic lupus erythematosus \|\| id:ebi-a-GCST003156 | Breast cancer (GWAS) \|\| id:ieu-a-1131 | ebi-a-GCST003156 | ieu-a-1131 | 32498 | rs2573219 | -0.0299428 | 0.05086877 | 0.55611033 |
| 18 | Systemic lupus erythematosus \|\| id:ebi-a-GCST003156 | Breast cancer (GWAS) \|\| id:ieu-a-1131 | ebi-a-GCST003156 | ieu-a-1131 | 32498 | rs268124 | -0.0037568 | 0.10679976 | 0.9719395 |
| 19 | Systemic lupus erythematosus \|\| id:ebi-a-GCST003156 | Breast cancer (GWAS) \|\| id:ieu-a-1131 | ebi-a-GCST003156 | ieu-a-1131 | 32498 | rs34703115 | -0.060696 | 0.08844732 | 0.4925627 |
| 20 | Systemic lupus erythematosus \|\| id:ebi-a-GCST003156 | Breast cancer (GWAS) \|\| id:ieu-a-1131 | ebi-a-GCST003156 | ieu-a-1131 | 32498 | rs35000415 | 0.01888439 | 0.04746617 | 0.69074115 |
| 21 | Systemic lupus erythematosus \|\| id:ebi-a-GCST003156 | Breast cancer (GWAS) \|\| id:ieu-a-1131 | ebi-a-GCST003156 | ieu-a-1131 | 32498 | rs35251378 | -0.1030875 | 0.08399725 | 0.2197201 |
| 22 | Systemic lupus erythematosus \|\| id:ebi-a-GCST003156 | Breast cancer (GWAS) \|\| id:ieu-a-1131 | ebi-a-GCST003156 | ieu-a-1131 | 32498 | rs353608 | 0.04722804 | 0.09391939 | 0.61506471 |
| 23 | Systemic lupus erythematosus \|\| id:ebi-a-GCST003156 | Breast cancer (GWAS) \|\| id:ieu-a-1131 | ebi-a-GCST003156 | ieu-a-1131 | 32498 | rs3747093 | 0.09338171 | 0.08537757 | 0.27406464 |
| 24 | Systemic lupus erythematosus \|\| id:ebi-a-GCST003156 | Breast cancer (GWAS) \|\| id:ieu-a-1131 | ebi-a-GCST003156 | ieu-a-1131 | 32498 | rs389884 | 0.02111571 | 0.0295189 | 0.4744061 |
| 25 | Systemic lupus erythematosus \|\| id:ebi-a-GCST003156 | Breast cancer (GWAS) \|\| id:ieu-a-1131 | ebi-a-GCST003156 | ieu-a-1131 | 32498 | rs4274624 | 0.0237663 | 0.03770443 | 0.52847755 |
| 26 | Systemic lupus erythematosus \|\| id:ebi-a-GCST003156 | Breast cancer (GWAS) \|\| id:ieu-a-1131 | ebi-a-GCST003156 | ieu-a-1131 | 32498 | rs4388254 | 0.16832437 | 0.11283282 | 0.13575072 |
| 27 | Systemic lupus erythematosus \|\| id:ebi-a-GCST003156 | Breast cancer (GWAS) \|\| id:ieu-a-1131 | ebi-a-GCST003156 | ieu-a-1131 | 32498 | rs4661543 | 0.07870659 | 0.1053065 | 0.45481922 |
| 28 | Systemic lupus erythematosus \|\| id:ebi-a-GCST003156 | Breast cancer (GWAS) \|\| id:ieu-a-1131 | ebi-a-GCST003156 | ieu-a-1131 | 32498 | rs4916215 | -0.1456459 | 0.08918008 | 0.10243412 |
| 29 | Systemic lupus erythematosus \|\| id:ebi-a-GCST003156 | Breast cancer (GWAS) \|\| id:ieu-a-1131 | ebi-a-GCST003156 | ieu-a-1131 | 32498 | rs58688157 | -0.0582583 | 0.09007636 | 0.51778329 |
| 30 | Systemic lupus erythematosus \|\| id:ebi-a-GCST003156 | Breast cancer (GWAS) \|\| id:ieu-a-1131 | ebi-a-GCST003156 | ieu-a-1131 | 32498 | rs58721818 | -0.0051709 | 0.08334347 | 0.95052796 |
| 31 | Systemic lupus erythematosus \|\| id:ebi-a-GCST003156 | Breast cancer (GWAS) \|\| id:ieu-a-1131 | ebi-a-GCST003156 | ieu-a-1131 | 32498 | rs6671847 | 0.10560671 | 0.08850848 | 0.23279817 |
| 32 | Systemic lupus erythematosus \|\| id:ebi-a-GCST003156 | Breast cancer (GWAS) \|\| id:ieu-a-1131 | ebi-a-GCST003156 | ieu-a-1131 | 32498 | rs6679677 | 0.25797095 | 0.09183528 | 0.00496862 |
| 33 | Systemic lupus erythematosus \|\| id:ebi-a-GCST003156 | Breast cancer (GWAS) \|\| id:ieu-a-1131 | ebi-a-GCST003156 | ieu-a-1131 | 32498 | rs6889239 | -0.0659146 | 0.07275818 | 0.3649673 |
| 34 | Systemic lupus erythematosus \|\| id:ebi-a-GCST003156 | Breast cancer (GWAS) \|\| id:ieu-a-1131 | ebi-a-GCST003156 | ieu-a-1131 | 32498 | rs7097397 | -0.162078 | 0.09874953 | 0.10073425 |
| 35 | Systemic lupus erythematosus \|\| id:ebi-a-GCST003156 | Breast cancer (GWAS) \|\| id:ieu-a-1131 | ebi-a-GCST003156 | ieu-a-1131 | 32498 | rs73068668 | -0.0451208 | 0.11534392 | 0.69566081 |
| 36 | Systemic lupus erythematosus \|\| id:ebi-a-GCST003156 | Breast cancer (GWAS) \|\| id:ieu-a-1131 | ebi-a-GCST003156 | ieu-a-1131 | 32498 | rs7768653 | 0.11351889 | 0.08598452 | 0.18676 |
| 37 | Systemic lupus erythematosus \|\| id:ebi-a-GCST003156 | Breast cancer (GWAS) \|\| id:ieu-a-1131 | ebi-a-GCST003156 | ieu-a-1131 | 32498 | rs7823055 | -0.0536136 | 0.05190257 | 0.30161933 |
| 38 | Systemic lupus erythematosus \|\| id:ebi-a-GCST003156 | Breast cancer (GWAS) \|\| id:ieu-a-1131 | ebi-a-GCST003156 | ieu-a-1131 | 32498 | rs7899626 | -0.0005485 | 0.10695363 | 0.9959083 |
| 39 | Systemic lupus erythematosus \|\| id:ebi-a-GCST003156 | Breast cancer (GWAS) \|\| id:ieu-a-1131 | ebi-a-GCST003156 | ieu-a-1131 | 32498 | rs9852014 | 0.05913851 | 0.06074991 | 0.33031738 |
| 40 | Systemic lupus erythematosus \|\| id:ebi-a-GCST003156 | Breast cancer (GWAS) \|\| id:ieu-a-1131 | ebi-a-GCST003156 | ieu-a-1131 | 32498 | All - Inverse variance weighted | 0.00164745 | 0.01187644 | 0.88967488 |
| 41 | Systemic lupus erythematosus \|\| id:ebi-a-GCST003156 | Breast cancer (GWAS) \|\| id:ieu-a-1131 | ebi-a-GCST003156 | ieu-a-1131 | 32498 | All - MR Egger | 0.03093015 | 0.02482172 | 0.22056301 |
| 1 | Sicca syndrome [Sj<c2><9a>gren] \|\| id:finn-b-M13_SJOGREN | Breast cancer (GWAS) \|\| id:ieu-a-1131 | finn-b-M13_SJOGREN | ieu-a-1131 | 32498 | rs2853986 | 0.00167655 | 0.03391798 | 0.96057689 |
| 2 | Sicca syndrome [Sj<c2><9a>gren] \|\| id:finn-b-M13_SJOGREN | Breast cancer (GWAS) \|\| id:ieu-a-1131 | finn-b-M13_SJOGREN | ieu-a-1131 | 32498 | rs35407265 | 0.12422023 | 0.09547057 | 0.19321178 |
| 3 | Sicca syndrome [Sj<c2><9a>gren] \|\| id:finn-b-M13_SJOGREN | Breast cancer (GWAS) \|\| id:ieu-a-1131 | finn-b-M13_SJOGREN | ieu-a-1131 | 32498 | rs496315 | 0.04747116 | 0.10514641 | 0.65164595 |
| 4 | Sicca syndrome [Sj<c2><9a>gren] \|\| id:finn-b-M13_SJOGREN | Breast cancer (GWAS) \|\| id:ieu-a-1131 | finn-b-M13_SJOGREN | ieu-a-1131 | 32498 | All - Inverse variance weighted | 0.01812203 | 0.03057939 | 0.55343408 |
| 5 | Sicca syndrome [Sj<c2><9a>gren] \|\| id:finn-b-M13_SJOGREN | Breast cancer (GWAS) \|\| id:ieu-a-1131 | finn-b-M13_SJOGREN | ieu-a-1131 | 32498 | All - MR Egger | -0.027167 | 0.06347194 | 0.74253684 |
| 1 | Systemic sclerosis \|\| id:finn-b-M13_SYSTSLCE | Breast cancer (GWAS) \|\| id:ieu-a-1131 | finn-b-M13_SYSTSLCE | ieu-a-1131 | 32498 | rs36030018 | 0.05704081 | 0.05649962 | 0.31269725 |
| 2 | Systemic sclerosis \|\| id:finn-b-M13_SYSTSLCE | Breast cancer (GWAS) \|\| id:ieu-a-1131 | finn-b-M13_SYSTSLCE | ieu-a-1131 | 32498 | All - Inverse variance weighted | NA | NA | NA |
| 3 | Systemic sclerosis \|\| id:finn-b-M13_SYSTSLCE | Breast cancer (GWAS) \|\| id:ieu-a-1131 | finn-b-M13_SYSTSLCE | ieu-a-1131 | 32498 | All - MR Egger | NA | NA | NA |
| 1 | Dermatopolymyositis \|\| id:finn-b-M13_DERMATOPOLY | Breast cancer (GWAS) \|\| id:ieu-a-1131 | finn-b-M13_DERMATOPOLY | ieu-a-1131 | 32498 | rs3131617 | 0.00412789 | 0.01996397 | 0.83619189 |
| 2 | Dermatopolymyositis \|\| id:finn-b-M13_DERMATOPOLY | Breast cancer (GWAS) \|\| id:ieu-a-1131 | finn-b-M13_DERMATOPOLY | ieu-a-1131 | 32498 | All - Inverse variance weighted | NA | NA | NA |
| 3 | Dermatopolymyositis \|\| id:finn-b-M13_DERMATOPOLY | Breast cancer (GWAS) \|\| id:ieu-a-1131 | finn-b-M13_DERMATOPOLY | ieu-a-1131 | 32498 | All - MR Egger | NA | NA | NA |
| 1 | Polymyositis \|\| id:finn-b-M13_POLYMYO | Breast cancer (GWAS) \|\| id:ieu-a-1131 | finn-b-M13_POLYMYO | ieu-a-1131 | 32498 | rs2596500 | -0.0032709 | 0.01469121 | 0.82381453 |
| 2 | Polymyositis \|\| id:finn-b-M13_POLYMYO | Breast cancer (GWAS) \|\| id:ieu-a-1131 | finn-b-M13_POLYMYO | ieu-a-1131 | 32498 | All - Inverse variance weighted | NA | NA | NA |
| 3 | Polymyositis \|\| id:finn-b-M13_POLYMYO | Breast cancer (GWAS) \|\| id:ieu-a-1131 | finn-b-M13_POLYMYO | ieu-a-1131 | 32498 | All - MR Egger | NA | NA | NA |
| 1 | Osteoarthritis of the hip or knee \|\| id:ebi-a-GCST007092 | Breast cancer (GWAS) \|\| id:ieu-a-1131 | ebi-a-GCST007092 | ieu-a-1131 | 32498 | rs10758594 | 0.33027523 | 0.40366972 | 0.41325338 |
| 2 | Osteoarthritis of the hip or knee \|\| id:ebi-a-GCST007092 | Breast cancer (GWAS) \|\| id:ieu-a-1131 | ebi-a-GCST007092 | ieu-a-1131 | 32498 | rs10948196 | -0.4413146 | 0.42253521 | 0.29627981 |
| 3 | Osteoarthritis of the hip or knee \|\| id:ebi-a-GCST007092 | Breast cancer (GWAS) \|\| id:ieu-a-1131 | ebi-a-GCST007092 | ieu-a-1131 | 32498 | rs11732213 | 0.29251701 | 0.37244898 | 0.43222604 |
| 4 | Osteoarthritis of the hip or knee \|\| id:ebi-a-GCST007092 | Breast cancer (GWAS) \|\| id:ieu-a-1131 | ebi-a-GCST007092 | ieu-a-1131 | 32498 | rs11923760 | 0.03348214 | 0.41294643 | 0.93537747 |
| 5 | Osteoarthritis of the hip or knee \|\| id:ebi-a-GCST007092 | Breast cancer (GWAS) \|\| id:ieu-a-1131 | ebi-a-GCST007092 | ieu-a-1131 | 32498 | rs11997261 | -0.4852399 | 0.38191882 | 0.2038954 |
| 6 | Osteoarthritis of the hip or knee \|\| id:ebi-a-GCST007092 | Breast cancer (GWAS) \|\| id:ieu-a-1131 | ebi-a-GCST007092 | ieu-a-1131 | 32498 | rs12470967 | 0.22708333 | 0.40416667 | 0.57421436 |
| 7 | Osteoarthritis of the hip or knee \|\| id:ebi-a-GCST007092 | Breast cancer (GWAS) \|\| id:ieu-a-1131 | ebi-a-GCST007092 | ieu-a-1131 | 32498 | rs143384 | 0.11356467 | 0.28864353 | 0.69399259 |
| 8 | Osteoarthritis of the hip or knee \|\| id:ebi-a-GCST007092 | Breast cancer (GWAS) \|\| id:ieu-a-1131 | ebi-a-GCST007092 | ieu-a-1131 | 32498 | rs17659798 | -0.554731 | 0.36549165 | 0.12907327 |
| 9 | Osteoarthritis of the hip or knee \|\| id:ebi-a-GCST007092 | Breast cancer (GWAS) \|\| id:ieu-a-1131 | ebi-a-GCST007092 | ieu-a-1131 | 32498 | rs2299285 | 0.25485961 | 0.39740821 | 0.52132496 |
| 10 | Osteoarthritis of the hip or knee \|\| id:ebi-a-GCST007092 | Breast cancer (GWAS) \|\| id:ieu-a-1131 | ebi-a-GCST007092 | ieu-a-1131 | 32498 | rs2472304 | 0.02654867 | 0.40486726 | 0.94771717 |
| 11 | Osteoarthritis of the hip or knee \|\| id:ebi-a-GCST007092 | Breast cancer (GWAS) \|\| id:ieu-a-1131 | ebi-a-GCST007092 | ieu-a-1131 | 32498 | rs2622873 | 0.00877193 | 0.38596491 | 0.98186782 |
| 12 | Osteoarthritis of the hip or knee \|\| id:ebi-a-GCST007092 | Breast cancer (GWAS) \|\| id:ieu-a-1131 | ebi-a-GCST007092 | ieu-a-1131 | 32498 | rs2820443 | -0.4953959 | 0.35543278 | 0.16338333 |
| 13 | Osteoarthritis of the hip or knee \|\| id:ebi-a-GCST007092 | Breast cancer (GWAS) \|\| id:ieu-a-1131 | ebi-a-GCST007092 | ieu-a-1131 | 32498 | rs2953013 | 0.4980916 | 0.36259542 | 0.16953974 |
| 14 | Osteoarthritis of the hip or knee \|\| id:ebi-a-GCST007092 | Breast cancer (GWAS) \|\| id:ieu-a-1131 | ebi-a-GCST007092 | ieu-a-1131 | 32498 | rs3774354 | -0.1548507 | 0.33955224 | 0.64835837 |
| 15 | Osteoarthritis of the hip or knee \|\| id:ebi-a-GCST007092 | Breast cancer (GWAS) \|\| id:ieu-a-1131 | ebi-a-GCST007092 | ieu-a-1131 | 32498 | rs3821262 | 0.42960289 | 0.31588448 | 0.17382992 |
| 16 | Osteoarthritis of the hip or knee \|\| id:ebi-a-GCST007092 | Breast cancer (GWAS) \|\| id:ieu-a-1131 | ebi-a-GCST007092 | ieu-a-1131 | 32498 | rs3884606 | -0.4965675 | 0.40961098 | 0.2254012 |
| 17 | Osteoarthritis of the hip or knee \|\| id:ebi-a-GCST007092 | Breast cancer (GWAS) \|\| id:ieu-a-1131 | ebi-a-GCST007092 | ieu-a-1131 | 32498 | rs4630744 | 0.15241636 | 0.32527881 | 0.639376 |
| 18 | Osteoarthritis of the hip or knee \|\| id:ebi-a-GCST007092 | Breast cancer (GWAS) \|\| id:ieu-a-1131 | ebi-a-GCST007092 | ieu-a-1131 | 32498 | rs4979341 | 0.27470687 | 0.33333333 | 0.40987103 |
| 19 | Osteoarthritis of the hip or knee \|\| id:ebi-a-GCST007092 | Breast cancer (GWAS) \|\| id:ieu-a-1131 | ebi-a-GCST007092 | ieu-a-1131 | 32498 | rs75621460 | 0.12869337 | 0.50558109 | 0.79907419 |
| 20 | Osteoarthritis of the hip or knee \|\| id:ebi-a-GCST007092 | Breast cancer (GWAS) \|\| id:ieu-a-1131 | ebi-a-GCST007092 | ieu-a-1131 | 32498 | rs7935877 | 0.45377129 | 0.43430657 | 0.29610713 |
| 21 | Osteoarthritis of the hip or knee \|\| id:ebi-a-GCST007092 | Breast cancer (GWAS) \|\| id:ieu-a-1131 | ebi-a-GCST007092 | ieu-a-1131 | 32498 | rs9277552 | 0.00675676 | 0.36486486 | 0.9852252 |
| 22 | Osteoarthritis of the hip or knee \|\| id:ebi-a-GCST007092 | Breast cancer (GWAS) \|\| id:ieu-a-1131 | ebi-a-GCST007092 | ieu-a-1131 | 32498 | rs9930333 | -0.9741379 | 0.39008621 | 0.01251651 |
| 23 | Osteoarthritis of the hip or knee \|\| id:ebi-a-GCST007092 | Breast cancer (GWAS) \|\| id:ieu-a-1131 | ebi-a-GCST007092 | ieu-a-1131 | 32498 | rs9977881 | 0.00164745 | 0.37891269 | 0.99653095 |
| 24 | Osteoarthritis of the hip or knee \|\| id:ebi-a-GCST007092 | Breast cancer (GWAS) \|\| id:ieu-a-1131 | ebi-a-GCST007092 | ieu-a-1131 | 32498 | All - Inverse variance weighted | -0.0041084 | 0.0792366 | 0.95864805 |
| 25 | Osteoarthritis of the hip or knee \|\| id:ebi-a-GCST007092 | Breast cancer (GWAS) \|\| id:ieu-a-1131 | ebi-a-GCST007092 | ieu-a-1131 | 32498 | All - MR Egger | 0.61265892 | 0.44738228 | 0.18533277 |
| 1 | Ankylosing spondylitis \|\| id:ebi-a-GCST005529 | Breast cancer (GWAS) \|\| id:ieu-a-1131 | ebi-a-GCST005529 | ieu-a-1131 | 32498 | rs1041926 | -0.6361162 | 0.81652726 | 0.43594983 |
| 2 | Ankylosing spondylitis \|\| id:ebi-a-GCST005529 | Breast cancer (GWAS) \|\| id:ieu-a-1131 | ebi-a-GCST005529 | ieu-a-1131 | 32498 | rs11190133 | 0.59349634 | 0.56101644 | 0.29010343 |
| 3 | Ankylosing spondylitis \|\| id:ebi-a-GCST005529 | Breast cancer (GWAS) \|\| id:ieu-a-1131 | ebi-a-GCST005529 | ieu-a-1131 | 32498 | rs11209026 | 0.29830862 | 0.34947482 | 0.39333156 |
| 4 | Ankylosing spondylitis \|\| id:ebi-a-GCST005529 | Breast cancer (GWAS) \|\| id:ieu-a-1131 | ebi-a-GCST005529 | ieu-a-1131 | 32498 | rs1128905 | -0.5650075 | 0.74209938 | 0.4464399 |
| 5 | Ankylosing spondylitis \|\| id:ebi-a-GCST005529 | Breast cancer (GWAS) \|\| id:ieu-a-1131 | ebi-a-GCST005529 | ieu-a-1131 | 32498 | rs11624293 | 1.18503589 | 0.72081907 | 0.10017353 |
| 6 | Ankylosing spondylitis \|\| id:ebi-a-GCST005529 | Breast cancer (GWAS) \|\| id:ieu-a-1131 | ebi-a-GCST005529 | ieu-a-1131 | 32498 | rs1250550 | 0.39176525 | 0.77200799 | 0.61183021 |
| 7 | Ankylosing spondylitis \|\| id:ebi-a-GCST005529 | Breast cancer (GWAS) \|\| id:ieu-a-1131 | ebi-a-GCST005529 | ieu-a-1131 | 32498 | rs12615545 | 0.20806507 | 0.69093307 | 0.76331051 |
| 8 | Ankylosing spondylitis \|\| id:ebi-a-GCST005529 | Breast cancer (GWAS) \|\| id:ieu-a-1131 | ebi-a-GCST005529 | ieu-a-1131 | 32498 | rs1801274 | 1.01114631 | 0.6912133 | 0.14350647 |
| 9 | Ankylosing spondylitis \|\| id:ebi-a-GCST005529 | Breast cancer (GWAS) \|\| id:ieu-a-1131 | ebi-a-GCST005529 | ieu-a-1131 | 32498 | rs2517655 | 0.00339806 | 0.23220095 | 0.98832406 |
| 10 | Ankylosing spondylitis \|\| id:ebi-a-GCST005529 | Breast cancer (GWAS) \|\| id:ieu-a-1131 | ebi-a-GCST005529 | ieu-a-1131 | 32498 | rs2531875 | 0.17567424 | 0.66609817 | 0.79198315 |
| 11 | Ankylosing spondylitis \|\| id:ebi-a-GCST005529 | Breast cancer (GWAS) \|\| id:ieu-a-1131 | ebi-a-GCST005529 | ieu-a-1131 | 32498 | rs2596501 | 0.19168155 | 0.11487764 | 0.09520235 |
| 12 | Ankylosing spondylitis \|\| id:ebi-a-GCST005529 | Breast cancer (GWAS) \|\| id:ieu-a-1131 | ebi-a-GCST005529 | ieu-a-1131 | 32498 | rs27529 | -0.0257917 | 0.29660484 | 0.93070607 |
| 13 | Ankylosing spondylitis \|\| id:ebi-a-GCST005529 | Breast cancer (GWAS) \|\| id:ieu-a-1131 | ebi-a-GCST005529 | ieu-a-1131 | 32498 | rs2836883 | -0.0655295 | 0.49903218 | 0.8955276 |
| 14 | Ankylosing spondylitis \|\| id:ebi-a-GCST005529 | Breast cancer (GWAS) \|\| id:ieu-a-1131 | ebi-a-GCST005529 | ieu-a-1131 | 32498 | rs35164067 | 0.38934294 | 0.76903276 | 0.6126628 |
| 15 | Ankylosing spondylitis \|\| id:ebi-a-GCST005529 | Breast cancer (GWAS) \|\| id:ieu-a-1131 | ebi-a-GCST005529 | ieu-a-1131 | 32498 | rs4129267 | 0.91977184 | 0.58501389 | 0.11589902 |
| 16 | Ankylosing spondylitis \|\| id:ebi-a-GCST005529 | Breast cancer (GWAS) \|\| id:ieu-a-1131 | ebi-a-GCST005529 | ieu-a-1131 | 32498 | rs41299637 | 0.39689957 | 0.49932527 | 0.42668808 |
| 17 | Ankylosing spondylitis \|\| id:ebi-a-GCST005529 | Breast cancer (GWAS) \|\| id:ieu-a-1131 | ebi-a-GCST005529 | ieu-a-1131 | 32498 | rs4672505 | -0.3161741 | 0.30613679 | 0.30170364 |
| 18 | Ankylosing spondylitis \|\| id:ebi-a-GCST005529 | Breast cancer (GWAS) \|\| id:ieu-a-1131 | ebi-a-GCST005529 | ieu-a-1131 | 32498 | rs4676410 | 1.48391183 | 0.78999623 | 0.06032942 |
| 19 | Ankylosing spondylitis \|\| id:ebi-a-GCST005529 | Breast cancer (GWAS) \|\| id:ieu-a-1131 | ebi-a-GCST005529 | ieu-a-1131 | 32498 | rs6556416 | 1.01526064 | 0.74954789 | 0.17557776 |
| 20 | Ankylosing spondylitis \|\| id:ebi-a-GCST005529 | Breast cancer (GWAS) \|\| id:ieu-a-1131 | ebi-a-GCST005529 | ieu-a-1131 | 32498 | rs6600247 | 0.74010453 | 0.53908848 | 0.16978923 |
| 21 | Ankylosing spondylitis \|\| id:ebi-a-GCST005529 | Breast cancer (GWAS) \|\| id:ieu-a-1131 | ebi-a-GCST005529 | ieu-a-1131 | 32498 | rs7191548 | -0.2321365 | 0.74844008 | 0.75643896 |
| 22 | Ankylosing spondylitis \|\| id:ebi-a-GCST005529 | Breast cancer (GWAS) \|\| id:ieu-a-1131 | ebi-a-GCST005529 | ieu-a-1131 | 32498 | rs743479 | 0.00426922 | 0.75992059 | 0.99551753 |
| 23 | Ankylosing spondylitis \|\| id:ebi-a-GCST005529 | Breast cancer (GWAS) \|\| id:ieu-a-1131 | ebi-a-GCST005529 | ieu-a-1131 | 32498 | rs9901869 | 0.6268885 | 0.54852744 | 0.25309791 |
| 24 | Ankylosing spondylitis \|\| id:ebi-a-GCST005529 | Breast cancer (GWAS) \|\| id:ieu-a-1131 | ebi-a-GCST005529 | ieu-a-1131 | 32498 | All - Inverse variance weighted | 0.1925947 | 0.07729278 | 0.01271136 |
| 25 | Ankylosing spondylitis \|\| id:ebi-a-GCST005529 | Breast cancer (GWAS) \|\| id:ieu-a-1131 | ebi-a-GCST005529 | ieu-a-1131 | 32498 | All - MR Egger | 0.0539244 | 0.13043141 | 0.68348219 |
| 1 | Diseases of the musculoskeletal system and connective tissue \|\| id:finn-b-M13_MUSCULOSKELETAL | Breast cancer (GWAS) \|\| id:ieu-a-1131 | finn-b-M13_MUSCULOSKELETAL | ieu-a-1131 | 32498 | rs113738740 | 0.56489362 | 0.73723404 | 0.44353722 |
| 2 | Diseases of the musculoskeletal system and connective tissue \|\| id:finn-b-M13_MUSCULOSKELETAL | Breast cancer (GWAS) \|\| id:ieu-a-1131 | finn-b-M13_MUSCULOSKELETAL | ieu-a-1131 | 32498 | rs11663824 | 0.58673469 | 0.44897959 | 0.19127447 |
| 3 | Diseases of the musculoskeletal system and connective tissue \|\| id:finn-b-M13_MUSCULOSKELETAL | Breast cancer (GWAS) \|\| id:ieu-a-1131 | finn-b-M13_MUSCULOSKELETAL | ieu-a-1131 | 32498 | rs148015908 | 0.31961259 | 0.25242131 | 0.20544612 |
| 4 | Diseases of the musculoskeletal system and connective tissue \|\| id:finn-b-M13_MUSCULOSKELETAL | Breast cancer (GWAS) \|\| id:ieu-a-1131 | finn-b-M13_MUSCULOSKELETAL | ieu-a-1131 | 32498 | rs4272793 | -0.0610687 | 0.45038168 | 0.89214287 |
| 5 | Diseases of the musculoskeletal system and connective tissue \|\| id:finn-b-M13_MUSCULOSKELETAL | Breast cancer (GWAS) \|\| id:ieu-a-1131 | finn-b-M13_MUSCULOSKELETAL | ieu-a-1131 | 32498 | All - Inverse variance weighted | 0.31595495 | 0.19095293 | 0.09800116 |
| 6 | Diseases of the musculoskeletal system and connective tissue \|\| id:finn-b-M13_MUSCULOSKELETAL | Breast cancer (GWAS) \|\| id:ieu-a-1131 | finn-b-M13_MUSCULOSKELETAL | ieu-a-1131 | 32498 | All - MR Egger | 0.36125253 | 0.33765003 | 0.39666971 |
