# Supplementary material for: Diseases of the musculoskeletal system and connective tissue and risk of breast cancer: Mendelian randomization study in European and East Asian populations
Source: Front Oncol. 2023 Apr 26;13:1170119. doi: 10.3389/fonc.2023.1170119 (PMC10169740; doi:10.3389/fonc.2023.1170119)
Supplement: Supplementary file 7 [file Table_2.docx]

Supplementary Tab. 2 Selected SNP in the East Asian population

|  | exposure | outcome | id.exposure | id.outcome | samplesize | SNP | b | se | p |
| --- | --- | --- | --- | --- | --- | --- | --- | --- | --- |
| 1 | Rheumatoid arthritis \|\| id:ieu-a-831 | Breast cancer \|\| id:bbj-a-160 | ieu-a-831 | bbj-a-160 | 95283 | rs10821944 | 0.04194796 | 0.12764415 | 0.74243384 |
| 2 | Rheumatoid arthritis \|\| id:ieu-a-831 | Breast cancer \|\| id:bbj-a-160 | ieu-a-831 | bbj-a-160 | 95283 | rs10946216 | -0.1143859 | 0.08198169 | 0.16293703 |
| 3 | Rheumatoid arthritis \|\| id:ieu-a-831 | Breast cancer \|\| id:bbj-a-160 | ieu-a-831 | bbj-a-160 | 95283 | rs11889341 | -0.3140703 | 0.13703791 | 0.0219143 |
| 4 | Rheumatoid arthritis \|\| id:ieu-a-831 | Breast cancer \|\| id:bbj-a-160 | ieu-a-831 | bbj-a-160 | 95283 | rs17427599 | 0.02148875 | 0.03247226 | 0.50812683 |
| 5 | Rheumatoid arthritis \|\| id:ieu-a-831 | Breast cancer \|\| id:bbj-a-160 | ieu-a-831 | bbj-a-160 | 95283 | rs2240339 | 0.00499234 | 0.10774325 | 0.96304285 |
| 6 | Rheumatoid arthritis \|\| id:ieu-a-831 | Breast cancer \|\| id:bbj-a-160 | ieu-a-831 | bbj-a-160 | 95283 | rs2244020 | -0.1331262 | 0.06296063 | 0.03447805 |
| 7 | Rheumatoid arthritis \|\| id:ieu-a-831 | Breast cancer \|\| id:bbj-a-160 | ieu-a-831 | bbj-a-160 | 95283 | rs2856821 | -0.0617981 | 0.08416736 | 0.46280903 |
| 8 | Rheumatoid arthritis \|\| id:ieu-a-831 | Breast cancer \|\| id:bbj-a-160 | ieu-a-831 | bbj-a-160 | 95283 | rs3734708 | -0.039018 | 0.11315135 | 0.73022208 |
| 9 | Rheumatoid arthritis \|\| id:ieu-a-831 | Breast cancer \|\| id:bbj-a-160 | ieu-a-831 | bbj-a-160 | 95283 | rs3819720 | -0.0830417 | 0.06530006 | 0.20348183 |
| 10 | Rheumatoid arthritis \|\| id:ieu-a-831 | Breast cancer \|\| id:bbj-a-160 | ieu-a-831 | bbj-a-160 | 95283 | rs449635 | -0.1526769 | 0.07076143 | 0.0309567 |
| 11 | Rheumatoid arthritis \|\| id:ieu-a-831 | Breast cancer \|\| id:bbj-a-160 | ieu-a-831 | bbj-a-160 | 95283 | rs58667488 | -0.0483524 | 0.03289475 | 0.14158579 |
| 12 | Rheumatoid arthritis \|\| id:ieu-a-831 | Breast cancer \|\| id:bbj-a-160 | ieu-a-831 | bbj-a-160 | 95283 | rs909267 | -0.2804585 | 0.06760852 | 3.35E-05 |
| 13 | Rheumatoid arthritis \|\| id:ieu-a-831 | Breast cancer \|\| id:bbj-a-160 | ieu-a-831 | bbj-a-160 | 95283 | rs9494892 | -0.2334243 | 0.12673064 | 0.06549079 |
| 14 | Rheumatoid arthritis \|\| id:ieu-a-831 | Breast cancer \|\| id:bbj-a-160 | ieu-a-831 | bbj-a-160 | 95283 | All - Inverse variance weighted | -0.0663727 | 0.02564025 | 0.00963632 |
| 15 | Rheumatoid arthritis \|\| id:ieu-a-831 | Breast cancer \|\| id:bbj-a-160 | ieu-a-831 | bbj-a-160 | 95283 | All - MR Egger | -0.0136705 | 0.05363902 | 0.80353282 |
| 1 | Systemic lupus erythematosus \|\| id:ebi-a-GCST90011866 | Breast cancer \|\| id:bbj-a-160 | ebi-a-GCST90011866 | bbj-a-160 | 95283 | rs10036748 | -0.1105804 | 0.10842306 | 0.30777708 |
| 2 | Systemic lupus erythematosus \|\| id:ebi-a-GCST90011866 | Breast cancer \|\| id:bbj-a-160 | ebi-a-GCST90011866 | bbj-a-160 | 95283 | rs10516487 | 0.03715308 | 0.12732627 | 0.77044388 |
| 3 | Systemic lupus erythematosus \|\| id:ebi-a-GCST90011866 | Breast cancer \|\| id:bbj-a-160 | ebi-a-GCST90011866 | bbj-a-160 | 95283 | rs1167791 | 0.01643584 | 0.1100843 | 0.88131509 |
| 4 | Systemic lupus erythematosus \|\| id:ebi-a-GCST90011866 | Breast cancer \|\| id:bbj-a-160 | ebi-a-GCST90011866 | bbj-a-160 | 95283 | rs11889341 | -0.1184205 | 0.05167027 | 0.0219143 |
| 5 | Systemic lupus erythematosus \|\| id:ebi-a-GCST90011866 | Breast cancer \|\| id:bbj-a-160 | ebi-a-GCST90011866 | bbj-a-160 | 95283 | rs12599402 | -0.011955 | 0.10972113 | 0.91323564 |
| 6 | Systemic lupus erythematosus \|\| id:ebi-a-GCST90011866 | Breast cancer \|\| id:bbj-a-160 | ebi-a-GCST90011866 | bbj-a-160 | 95283 | rs13213165 | 0.00804256 | 0.07013019 | 0.90869849 |
| 7 | Systemic lupus erythematosus \|\| id:ebi-a-GCST90011866 | Breast cancer \|\| id:bbj-a-160 | ebi-a-GCST90011866 | bbj-a-160 | 95283 | rs13385731 | -0.0197888 | 0.07896251 | 0.80211579 |
| 8 | Systemic lupus erythematosus \|\| id:ebi-a-GCST90011866 | Breast cancer \|\| id:bbj-a-160 | ebi-a-GCST90011866 | bbj-a-160 | 95283 | rs16869875 | -0.0793586 | 0.06691125 | 0.23561158 |
| 9 | Systemic lupus erythematosus \|\| id:ebi-a-GCST90011866 | Breast cancer \|\| id:bbj-a-160 | ebi-a-GCST90011866 | bbj-a-160 | 95283 | rs16870693 | 0.12362406 | 0.06855053 | 0.07132535 |
| 10 | Systemic lupus erythematosus \|\| id:ebi-a-GCST90011866 | Breast cancer \|\| id:bbj-a-160 | ebi-a-GCST90011866 | bbj-a-160 | 95283 | rs201036579 | -0.2593013 | 0.14109764 | 0.06610023 |
| 11 | Systemic lupus erythematosus \|\| id:ebi-a-GCST90011866 | Breast cancer \|\| id:bbj-a-160 | ebi-a-GCST90011866 | bbj-a-160 | 95283 | rs244689 | -0.0217347 | 0.14445329 | 0.88040012 |
| 12 | Systemic lupus erythematosus \|\| id:ebi-a-GCST90011866 | Breast cancer \|\| id:bbj-a-160 | ebi-a-GCST90011866 | bbj-a-160 | 95283 | rs2618473 | -0.1250869 | 0.06042591 | 0.03844416 |
| 13 | Systemic lupus erythematosus \|\| id:ebi-a-GCST90011866 | Breast cancer \|\| id:bbj-a-160 | ebi-a-GCST90011866 | bbj-a-160 | 95283 | rs2841281 | -0.0673549 | 0.10768753 | 0.53166526 |
| 14 | Systemic lupus erythematosus \|\| id:ebi-a-GCST90011866 | Breast cancer \|\| id:bbj-a-160 | ebi-a-GCST90011866 | bbj-a-160 | 95283 | rs3800387 | -0.07897 | 0.1228394 | 0.52030743 |
| 15 | Systemic lupus erythematosus \|\| id:ebi-a-GCST90011866 | Breast cancer \|\| id:bbj-a-160 | ebi-a-GCST90011866 | bbj-a-160 | 95283 | rs4134466 | -0.0152534 | 0.09700562 | 0.87505402 |
| 16 | Systemic lupus erythematosus \|\| id:ebi-a-GCST90011866 | Breast cancer \|\| id:bbj-a-160 | ebi-a-GCST90011866 | bbj-a-160 | 95283 | rs41430444 | -0.0270929 | 0.08801267 | 0.75821192 |
| 17 | Systemic lupus erythematosus \|\| id:ebi-a-GCST90011866 | Breast cancer \|\| id:bbj-a-160 | ebi-a-GCST90011866 | bbj-a-160 | 95283 | rs451263 | -0.1462893 | 0.12274992 | 0.23335263 |
| 18 | Systemic lupus erythematosus \|\| id:ebi-a-GCST90011866 | Breast cancer \|\| id:bbj-a-160 | ebi-a-GCST90011866 | bbj-a-160 | 95283 | rs4731532 | 0.01819911 | 0.07039962 | 0.79601218 |
| 19 | Systemic lupus erythematosus \|\| id:ebi-a-GCST90011866 | Breast cancer \|\| id:bbj-a-160 | ebi-a-GCST90011866 | bbj-a-160 | 95283 | rs4930642 | -0.0783121 | 0.11115111 | 0.48108721 |
| 20 | Systemic lupus erythematosus \|\| id:ebi-a-GCST90011866 | Breast cancer \|\| id:bbj-a-160 | ebi-a-GCST90011866 | bbj-a-160 | 95283 | rs5029937 | -0.1013071 | 0.0553986 | 0.06744533 |
| 21 | Systemic lupus erythematosus \|\| id:ebi-a-GCST90011866 | Breast cancer \|\| id:bbj-a-160 | ebi-a-GCST90011866 | bbj-a-160 | 95283 | rs55701306 | 0.02982834 | 0.12925692 | 0.81749517 |
| 22 | Systemic lupus erythematosus \|\| id:ebi-a-GCST90011866 | Breast cancer \|\| id:bbj-a-160 | ebi-a-GCST90011866 | bbj-a-160 | 95283 | rs620088 | -0.0897715 | 0.11096477 | 0.41850984 |
| 23 | Systemic lupus erythematosus \|\| id:ebi-a-GCST90011866 | Breast cancer \|\| id:bbj-a-160 | ebi-a-GCST90011866 | bbj-a-160 | 95283 | rs6941485 | 0.14042232 | 0.10292625 | 0.17247307 |
| 24 | Systemic lupus erythematosus \|\| id:ebi-a-GCST90011866 | Breast cancer \|\| id:bbj-a-160 | ebi-a-GCST90011866 | bbj-a-160 | 95283 | rs6993775 | -0.0189161 | 0.07225243 | 0.79347118 |
| 25 | Systemic lupus erythematosus \|\| id:ebi-a-GCST90011866 | Breast cancer \|\| id:bbj-a-160 | ebi-a-GCST90011866 | bbj-a-160 | 95283 | rs7097397 | -0.1969602 | 0.0897195 | 0.02814292 |
| 26 | Systemic lupus erythematosus \|\| id:ebi-a-GCST90011866 | Breast cancer \|\| id:bbj-a-160 | ebi-a-GCST90011866 | bbj-a-160 | 95283 | rs7486387 | -0.269403 | 0.12162566 | 0.02675886 |
| 27 | Systemic lupus erythematosus \|\| id:ebi-a-GCST90011866 | Breast cancer \|\| id:bbj-a-160 | ebi-a-GCST90011866 | bbj-a-160 | 95283 | rs7650774 | -0.1254488 | 0.1215723 | 0.30212524 |
| 28 | Systemic lupus erythematosus \|\| id:ebi-a-GCST90011866 | Breast cancer \|\| id:bbj-a-160 | ebi-a-GCST90011866 | bbj-a-160 | 95283 | rs9387400 | 0.2272302 | 0.11827267 | 0.05470143 |
| 29 | Systemic lupus erythematosus \|\| id:ebi-a-GCST90011866 | Breast cancer \|\| id:bbj-a-160 | ebi-a-GCST90011866 | bbj-a-160 | 95283 | All - Inverse variance weighted | -0.0502044 | 0.01820566 | 0.00582223 |
| 30 | Systemic lupus erythematosus \|\| id:ebi-a-GCST90011866 | Breast cancer \|\| id:bbj-a-160 | ebi-a-GCST90011866 | bbj-a-160 | 95283 | All - MR Egger | -0.066042 | 0.05328076 | 0.22622893 |
